# Game-thinking; utilizing serious games and gamification in nursing education – a systematic review and meta-analysis

**DOI:** 10.1186/s12909-024-06531-7

**Published:** 2025-01-29

**Authors:** Mats Nylén-Eriksen, Marko Stojiljkovic, Daniela Lillekroken, Katrin Lindeflaten, Elisabeth Hessevaagbakke, Tone Nygaard Flølo, Olav Johannes Hovland, Ada Marie Svarstad Solberg, Sylvia Hansen, Ann Kristin Bjørnnes, Christine Tørris

**Affiliations:** Institute of Nursing and Health Promotion, Oslo Metropolitan University, Oslo, Norway

**Keywords:** Game-thinking, Serious games, Game-based learning, Gamification, Digital media for learning, Video Games, Nursing, Higher education

## Abstract

**Background:**

The digital shift in higher education is moving from teacher-focused models to active learning with digital technologies, including the integration of game-based learning strategies. We aim to identify, assess, and summarize the findings of evidence and determine the effectiveness of game-thinking on learning outcomes in nursing education.

**Methods:**

A comprehensive search for relevant literature was conducted between April and May 2022 Seven databases ERIC, Scopus, ProQuest Education Source, MEDLINE, CINAHL, Web of Science, and Embase were utilized to locate original, peer-reviewed papers published in English. The review was conducted and reported in compliance with the Preferred Reporting Items for Systematic Reviews and Meta-Analyses (PRISMA) 2020 guidelines.

**Results:**

Overall, 3302 studies were initially screened based on their titles and abstract. From this selection 281 studies were then assessed for full-text eligibility. In the end, 70 studies, consisting of 27 Randomized Controlled Trials (RCTs) and 43 Quasi-experimental studies were included in the review. These studies encompassed data from a total of 8348 participants. The results from the narrative synthesis of the results revealed consistencies across the included studies and their findings. The meta-analysis suggested that game-thinking could be beneficial in nursing education, notably improving students’ academic achievement (Pooled ES = 0.99, [95%CI 0.53, 1.44]). The most significant effect of game-thinking on academic achievement was observed in the academic knowledge performance of nursing students (Pooled ES = 1.06, [95%CI 0.55, 1.57]), followed by academic skill performance (Pooled ES = 0.54, [95%CI 0.06, 1.03]).

**Conclusions:**

The systematic review and meta-analysis provide evidence supporting the effectiveness of game-thinking in nursing education. The findings highlight the potential of game-based learning in enhancing nursing education through knowledge acquisition, albeit with a nuanced effect on skill development. As nursing education continues to adapt to the digital era, integration of game-thinking strategies could serve as a valuable method for creating engaging and effective learning experiences for nursing students.

**Supplementary Information:**

The online version contains supplementary material available at 10.1186/s12909-024-06531-7.

## Introduction

The use of game-based methods [1] as a learning strategy in higher education is a part a digital transformation [2]. Game-thinking [3] have been introduced as umbrella term, encompassing two academically accepted game-based methods: gamification and serious games (SG) [4, 5]. To better understand game-thinking as a pedagogical strategy to improve nursing students´ academic achievement or perceptions [5], by redesigning traditional learning content [1, 6], utilizing either gamification or SG.

SG are full complete games that use all game design elements in various degrees for non-entertainment purposes, with the aim of enhancing student learning outcomes [5, 7–9], Gamification, however, applies one or a combination of game design elements in non-gaming contexts, like education, to influence and/or to impact learning outcomes [5, 8, 10–14]. Unlike SG, which intend to create a complete game [15], gamification uses specific game design elements without intending to create a game [8]. Game design elements are individual characteristics of games, with a significant role in the game play [10].

SG, seen by some as a sub-set of gamification, are created by gamifying traditional learning content, though this isn´t a universally held view [1, 11, 13]. Both SG and gamification uses the same game design elements, such as points, levels, avatars and leaderboards, to enhance student learning outcomes [5, 6, 10, 13]. However, the vast variety of these elements and the lack of standard classification system pose challenges across and within different research fields [5, 7–11, 15]. A proposed framework categorizes these elements into nine attribute categories, aiming to consolidate research and facilitate comparisons between studies [5, 16]. A revised definition of gamification are suggested replacing game design elements with the attributes categories [5]. A recent review on gamification focused on the attributes categories as recommended to improve health professions education [15].

Despite their differences, both SG and gamification should be included in systematic reviews, as they utilize the same game attribute categories in educational context, as they both represent the pedagogical design strategy of game-thinking [5]. This approach is echoed by other systematic reviews on educating health professionals, which include both SG and gamification in their reviews [7, 8]. Despite challenges like lack of consensus and uniformity [5, 7–11, 15, 17], research suggests that whether it´s a SG or gamification it has potential to enhance education by increasing student engagement, which could improve learning outcomes [14]. Nurse education is a complex process, unfolding across various arenas, both within educational institutions and in different professional practical contexts [18–20]. Game-thinking could be particularly beneficial for nursing students, who often start their studies with a naive view of the profession and struggle with their intricate learning journey, such as applying bioscience knowledge in their clinical practice [21, 22]. While games or game elements seem to increase students´ engagement and satisfaction by increasing enjoyment, still, research on their actual impact on learning in a nursing context is limited [22].

Engaging nursing students academically is challenging but crucial to enhance their performance across all aspects of the education [22, 23]. Educators should integrate various learning strategies and activities, both analog and digital, to maintain student engagement [18, 20, 24]. Academic underperformance, a key factor in involuntary attrition from nursing programs [25], should be addressed with interventions designed to boost academic performance [20]. Diverse teaching activities can help students navigate the complex learning environment [18, 20] by increasing enjoyment and engagement [22], and linking theory with practice [18]. Digital simulation games, or SG, Offer a potential intervention to increase engagement and enjoyment, and help students contextualize theory [7].

Addressing the global nursing shortage requires counteracting academic underperformance and enhancing professional competence [24]. Current research indicates that gamification and SG may improve the quality of health professions education [7–9, 13, 26, 27]. Integrating game-thinking strategies could enhance education quality in health professions. However, the impact on nursing students is not fully understood, underscoring the particular importance of research such as the present study and highlighting the needs for further research.

We aim to identify, assess, and summarize the effectiveness of game-thinking in nursing education, focusing on student learning outcomes and perceptions. To ensure a comprehensive understanding, our study is not based on a single theoretical framework due to the lack of a unifying theory for game-thinking, though motivation and engagement are central, and Self-determination theory (SDT) plays a significant role in game design and gamified experiences [28].

## Materials and methods

The procedure for this review was carried out in accordance with the PRISMA guidelines for systematic review and meta-analysis reporting [29]. The PRISMA statement is provided in Supplementary Materials File (S1)*.* The study was registered in the PROSPERO register (CRD42022324968).

### Eligibility criteria

We included studies centered on undergraduate nursing students, with interventions involving gamification or SG, employing randomized controlled trials (RCT) and quasi-experimental studies and with learning- and perception outcomes. We excluded studies that involved non-student nurses, interventions that were unrelated to gamification or SG, employed non-intervention or solely qualitative designs and outcomes unrelated to the students' learning or perceptions. However, we included mixed-method studies if data extraction was feasible. The full selection criteria are shown in Table 1.

**Table 1:** Inclusion/Exclusion criteria

	**Included**	**Excluded**
Concept	Gamification, serious games	Online teaching, online courses
Study design	Randomized controlled trials, Quasi-experimental, Pre-post evaluation	Grey literature, non-intervention studies, Qualitative methods
Targeted field	Nursing education (bachelor nursing programs)	Nurses post-graduation
Outcome	Nursing Students` Academic Achievement; - Academic Knowledge Performance such as; grades and exam/quiz scores - Academic Skill Performance such as; scoring on clinical observational skills, and scoring on the performance of a clinical procedure Nursing students' perceptions such as; self-efficacy or motivation/engagement/ student satisfaction using different measurement scales	Knowledge description without assessment of effect
Language	English, Scandinavian	All other language
Publication date	2010–2022	Published < 2010

### Information sources and search strategies

Studies published on the effects of gamification intervention or SG interventions in populations of nursing students were identified by performing systematic searches in the following databases: ERIC, Scopus, ProQuest Education Source, MEDLINE, CINAHL, Web of Science, and Embase. The searches were individually adapted to each database, and a combination of Medical Subject Headings (MeSH) and text word terms were employed in accordance with the database thesaurus.

The search strategies were developed with guidance from a health science librarian, and search terms according to Gamification and Nursing education were utilized (Table 2). The searches were performed in April–May 2022.

**Table 2:** Search strategy MEDLINE

Main term	Sub terms
Gamification	**MeSH:** Gamification, Learner-Generated Digital Media (LGDM), digital media literacies, digital media for learning, learner-generated digital media, Games Experimental, Video Games, Game Theory,
Nursing education	**MeSH:** Students, Nursing/OR Education, Nursing/OR Education, Nursing, Associate/OR Education, Nursing, Baccalaureate/OR Education, Nursing, Diploma Programmes/OR Nursing Education Research/ **Keywords:** Nurs* adj3 (stud* or educat*or bac* or programme*).tw,kf

If too many hits, include: MeSH: Educational Measurement/OR Academic Performance/OR Academic Failure/OR Student Dropouts/OR Self-efficacy/

Keywords: experience*. tw,kf. OR satisfaction*. tw,kf. OR academic* adj3(achieve* or progress* or withdraw* or persistence)

### Selection process

We used Covidence systematic review software [30] to perform the screening, eligibility assessment, data extraction, and quality assessment, the identified studies were uploaded to the online software. Duplicates were mostly removed by the software, and some were removed manually.

The included abstracts were independently double-screened against the eligibility criteria by the review authors, followed by an independently double re-assessing/screening of full-text articles performed by the two pre-selected reviewers. Finally, after exclusion, 70 studies were included in the systematic review.

**Fig. 1:**
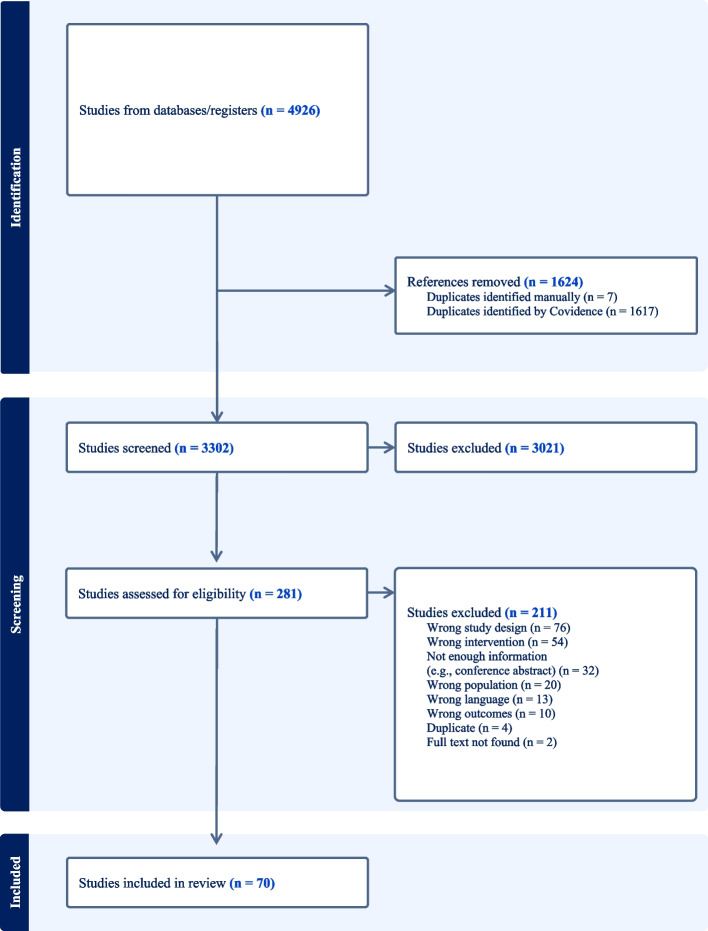
Flowchart – PRISMA

All conflicts in either abstract- or full text screening were handled by two pre-selected reviewers with the most experience with systematic reviews and meta-analysis. The flow diagram of the review process is shown in Fig. 1.

### Data collection process

Following the PRISMA guideline using forms with detailed instruction manuals in Covidence, prepared by, all authors contributed to the data extraction. First time authors received calibration exercise and guidance. The following data extracted from all included studies were displayed in a summary table and included; Title, Year Published, Country the Study was Conducted, Aim, Study Design, Sex, Age, Population Size (total number and portion of nursing students), Number of Groups Compared, Academic Year/Semester, Context/Course, Information About the Intervention, Type of game-thinking (e.g., gamification or SG), Outcome Measured, Results, Effect, and Conclusion.

### Data items

#### Outcomes

The primary outcome was Nursing Students' Academic Achievement (academic achievement), representing our purposefully broad approach to gain a comprehensive understanding of game-thinking, not focusing on specific measurement or learning outcomes, acknowledging the variety of ways to assess students’ academic progress. In studies within the same research domain but using different scales or outcomes, it falls on the researcher to determine if their combination yields a meaningful interpretation [31]. We further categorized our primary outcome into “Academic Knowledge Performance (knowledge performance)”, and “Academic Skill Performance (skill performance)”, to allow more detailed analysis of each subcategory.

The same procedure applies to our secondary outcomes, which are nursing students’ perceptions. Nursing students` perceptions (perceptions), were categorized into “motivation/engagement”, “self-efficacy”, “student satisfaction”, and “mix”.

Primary and secondary outcome categories reported in the retrieved studies are depicted in the summary table (Table 5).

### Other variables

Additionally, we extracted data relating to study-, participant- and intervention characteristics. 1.) Study characteristics included “publication year”, “country in which the study was conducted”, “study design” and “number of compared groups”. 2.) Participant characteristics included “sample size”, “sex”, “age”, “academic year; semester”, “context; courses (e.g., clinical practice, clinical skills lab, theoretical, mixed)”. 3.) Intervention characteristics included “type of game-thinking”, “intervention; analogue and/or digital” and “intervention; individual and/or team”.

### Study risk of bias assessment

The independent double-quality assessment was performed by all the reviewers, using the Joanna Briggs Institute (JBI) checklist for randomized controlled trials (RCT) [32] and quasi-experimental studies [33], as appropriate. The well-established JBI appraisal checklist for RCT´ (13 items), and the quasi-experimental checklist (9 items) assess internal validity, and are frequently used globally [34]. Any conflicts between the reviewers in the quality assessment were resolved in a consensus meeting.

### Effect measures

For continuous data the standardized mean difference (SMD) was calculated using the Practical Meta-Analysis Effect Size Calculator [35] which is a recommended and reliable online calculator [36]. If only median, range and sample size were provided, we estimated the mean and SD [37] to calculate the SMD. Effect size was expressed as Cohen´s *d*. Generally, a Cohen's *d* of 0.2–0.4 is considered a small effect size, 0.5–0.7 is considered a moderate effect size, and 0.8 or higher is considered a large effect size [36].

### Data synthesis and analysis

#### Preparation of data; serious games or gamification?

Classifying studies into SG or gamification was challenging due to inconsistent or lacking definitions. To minimize subjectivity studies with clearly defined interventions were grouped accordingly, while others were categorized based on the authors´ descriptions and their intended purpose of the game intervention which is a key factor distinguishing SG from gamified platforms or applications [15].

#### Narrative synthesis

Narrative summary presenting most central findings based on the characteristics and findings from included studies in text and tables.

#### Meta-analysis

We conducted three separate meta-analyses, because our primary outcome " Academic Achievement," encompassed both " Knowledge Performance" and " Skill Performance.". Considering game-thinking might impact knowledge and skill performance differently, we conducted separate analysis for each. In the meta-analysis of “Academic Achievement”, some studies included both knowledge and skill outcomes, while others had one. In dealing with effect size multiplicity, we followed recommendations [38] and performed a selection using a decision rule; “select the outcome prioritized by the authors of the specific study”, thus we avoided double counting and tried to reduce the risk of bias due to selection favoring our analysis.

The meta-analysis was conducted using the open-source statistics program JASP [39], and a restricted ML method, including both fixed and random effects by performing the Omnibus test of Model Coefficients and test of residual Heterogeneity was used to make our model.

A significant p-value on the Omnibus test of Model Coefficients represents effect of the intervention and suggests that the intervention has significant impact on the outcome being studied. A high significant p-value on the Test of Residual Heterogeneity indicated substantial variability, as this test assessed the variability remaining in the model after accounting for both fixed and random effects, which might be attributed to different effects of the intervention across the included studies [31]. The *I*^*2*^ was used to interpret the percentage of heterogeneity, i.e., the part of total variation resulting from between-studies variance [40], and based on the values considered to be low (< 25%), moderate (26–75%), or substantial (> 75%) [31].

Collectively, these findings offered evidence supporting the potential effectiveness, or lack thereof, of game-thinking. Results was based on comparison to the control group in the analyzed RCTs and/or quasi-experimental studies.

### Reporting bias assessment

The presence of substantial variability or heterogeneity in the meta-analysis was further checked by using Egger´s test and the PET-PEESE to test for publication bias and a p-value of > 0.05 indicates that there was no statistically significant evidence of publication bias [41, 42].

### Certainty assessment

The strength and quality of evidence for each included study were assessed based on the total scores on the Joanna Briggs Institute (JBI) critical appraisal checklists to assess the risk of bias. Based on previous systematic reviews utilizing JBI checklists, a study was categorized as low quality when scoring < 50%, moderate quality with scores ≥ 50–70% and high when scoring > 70% [43–45].

## Results

### Literature search results and general characteristics of the included studies

#### Literature search results

Initially, after removing duplicates, 3302 studies were screened by title and abstract. Subsequently, 281 studies were assessed for full-text eligibility; This process resulted in the inclusion of 70 studies, which underwent narrative synthesis and statistical analysis as shown in Fig. 1.

#### General characteristics of the included studies

The included studies (*N* = 70) consisted of 26 RCTs (37%), and 44 Quasi-experimental studies (63%) encompassing data from a total of 8348 participants, ranging from 10 participants [46] to 844 participants [47]. The population of men ranged from 0–61%, averaging at 19% (i.e., 1586 men). Asia contributed with 50% (*n* = 35) of the published articles, followed by Europe with 23% (*n* = 16), and North America with 17% (*n* = 12).

**Fig. 2:**
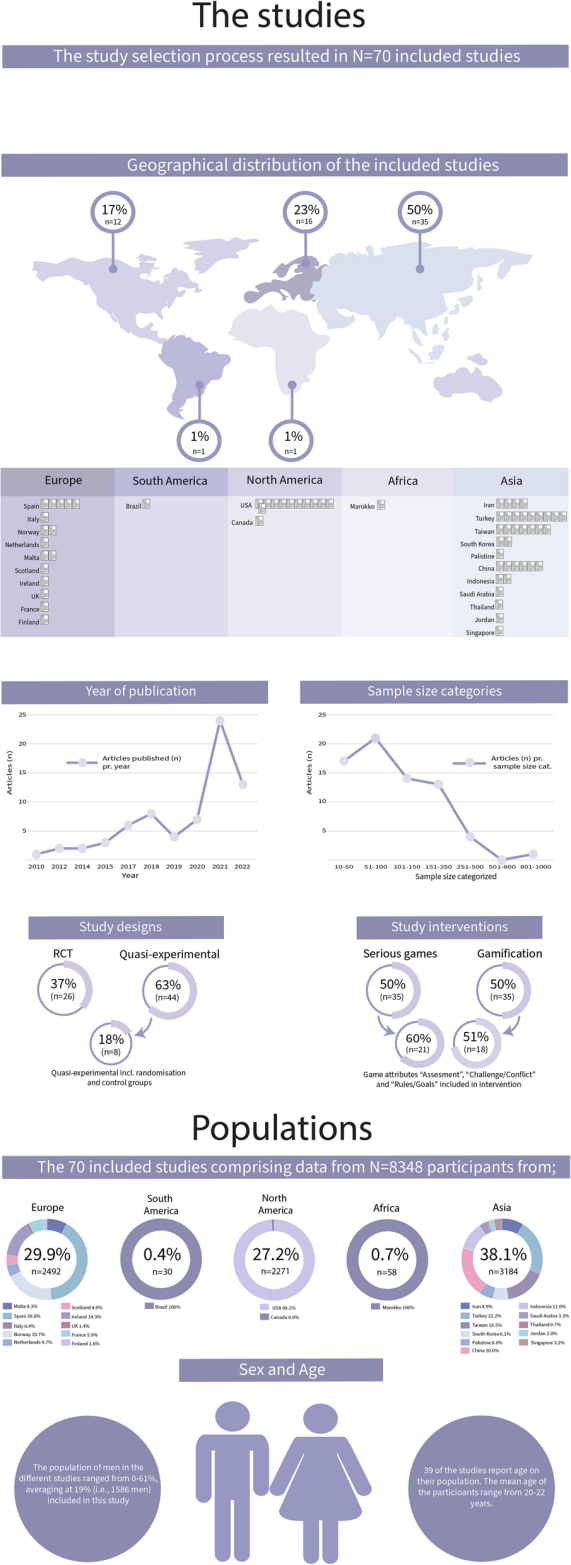
Visualization: general characteristics of the included studies

The year of publication ranged from 2010 to 2022, and interest in game-thinking appears to have grown in the subsequent years, culminating in the publication of 24 studies in 2021. The majority (96%, *n* = 67) of the studies included one or more outcomes on “Academic Achievement”, either exclusively (73%, *n* = 51) or in combination with one or more outcomes on “Perceptions” (23%, *n* = 16). These general characteristics of the included studies are visualized in Fig. 2. Additionally, 23% (*n* = 16) of the articles did not specify the academic year of the student participants. Among the remaining studies, 22% (n = 16) involved students in their final year of studies, while 20% (*n* = 13) exclusively focused on freshmen students.

### Narrative synthesis

Our systematic review (*N* = 70) on game-thinking reveals two distinct intervention categories equal in numbers: studies assessed as focusing on SG (*n* = 35) and studies focusing on gamification (*n* = 35). Of the studies on gamification, 94% (*n* = 33) included one or more outcomes on “Academic Achievement”, and similarly, the outcomes were included in 97% (*n* = 34) of the studies on SG. Interestingly, 85% (*n* = 28) of the studies on gamification and 82% (*n* = 28) of the studies on SG reported an effect on the academic achievement (refer to Table 3 for more details). See Table 4 for information related to the perceptions, please refer to Table 4. Only 14%, (*n* = 10) (i.e., four studies on gamification and six studies on SG) reported no effect on any of the outcomes measured, of which nine studies measuring academic achievement only [48–55] and two studies [56, 57] measuring only students’ perceptions. Table 5 provides a summary of all included studies.

**Table 3:**
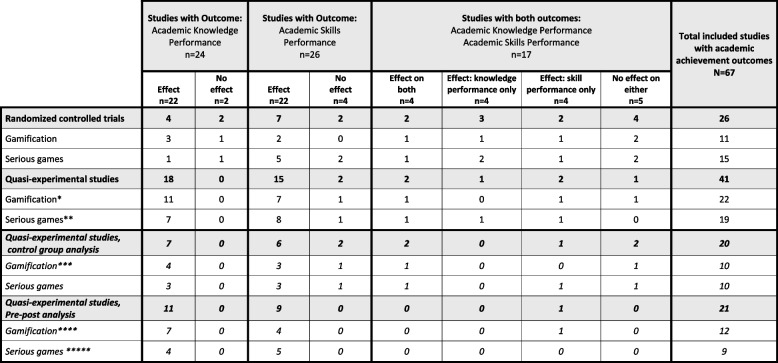
Academic achievement; effect of gamification and serious game interventions

The three missing studies includes only student perception outcomes. *2 missing. **1 missing, ***1 missing, ****1 missing, *****1 missing

**Table 4:**
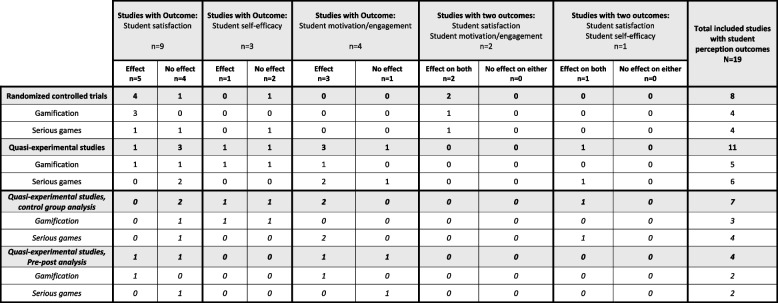
Student perceptions; effect of gamification and serious game interventions

**Table 5:** Summary table

**Author Year Country**	**Population**	**Context**	**Game-thinking**	**Game attribute categories**	**Game elements**	**Interventions**	**Comparison**	**Outcome**	**Results**
Aljezawi and Albashtawy (2015) [58] Jordan	Total 66, Intervention 34, Control 32. 8th semester, 39% male	Theoretical: Nursing Management and Ethics	Serious Game	Assessment, Conflict/challenge, Human interaction, Rules/goals	Points, Price for winning team, Competition, Teamwork, Goal attainment	Jeopardy style game: Teaching in quiz game format; Analogue; Team	Traditional didactic lecture	Academic knowledge performance, Student satisfaction	Statistically significant difference in academic knowledge performance and student satisfaction in favor of intervention group
Bayram and Caliskan (2019) [59] Turkey	Total 86, Intervention 43, Control 43. 1st year	Theoretical and skills performance: Fundaments of Nursing (Tracheostomy care)	Gamification	Action language, Assessment, Conflict/challenge, Game fiction, Environment, Immersion, Rules/goals	Progression, Challenge, Immersive virtual environment, Scenario, Goal attainment	Game-based virtual reality phone application: Theoretical and laboratory class with OSCE test; Digital; Both individual and team	Traditional didactic lecture with laboratory skills class and an OSCE test	Academic knowledge performance, Academic skills performance	Statistically significant difference in academic skills performance in favor of intervention group, however not in academic knowledge performance
Berg and Steinsbekk (2021) [48] Norway	Total 289, Intervention 146, Control 143. 1st year, 15% male	Skills performance: ABCDE	Gamification	Action language, Assessment Conflict/challenge, Environment, Immersion, Human interaction, Control, Rules/goals	VR-equipment, Feedback, Challenge, Cooperation, Avatars, Immersive virtual reality environment, Goal attainment	Virtual reality application ABCDE: Digital; Team	Practicing on physical equipment (mannequins)	Academic knowledge performance, Academic skills performance, Student motivation	No statistically significant difference in academic knowledge performance, nor academic skills performance in favor of intervention group
Blanié et al. (2020) [60] France	Total 146, Intervention 73, Control 73. 2nd year, 15% male	Theoretical: post-operative nursing	Serious Game	Action language, Assessment, Conflict/challenge, Environment, Game fiction, Immersion, Rules/goals	Points, Feedback, Adaptive challenge, Scenario, Immersive virtual environment, Goal attainment	Serious games simulation: Played a serious game consisting of two cases followed by debriefing; Analogue and digital; Individual and team	Traditional teaching: Case studies in paper form followed by a teaching course with a PowerPoint presentation	Academic skills performance, Student satisfaction	No statistically significant difference in academic skills performance, but in student satisfaction and motivation in favor of intervention group
Calik and Kapucu (2022) [61] Turkey	Total 60, Intervention 30, control 30. Avr. age 20, 2nd year, 7% male	Skills performance: Endocrine lesson, clinical practice	Serious Game	Action language, Assessment, Conflict/challenge, Environment, Game fiction, Immersion, Rules/goals	Points, Competitive scoring, Immersive virtual environment, Avatar, Scenario, Goal attainment	Serious game about diabetic ketoacidosis: Played a serious game after their 1st week of clinical practice; Digital; Individual	Standard clinical practice	Academic skills performance	Statistically significant difference in academic skills performance in favor of intervention group
Chang et al. (2021) [62] Taiwan	Total 100, Intervention 50, Control 50. Age 18-20 yrs., 0% male	Theoretical and Skills performance: Medication administration and nasotracheal suction	Gamification	Action language, Assessment, Conflict/challenge, Environment, Game fiction, Immersion, Rules/goals	Progression, Challenge, Time pressure, Immersive virtual environment, Scenario, Goal attainment	Virtual simulation based mobile learning app: Scenarios where students interact with the mobile phone application; Digital; Individual	Five scenarios written on paper in which different nursing activities were required	Academic knowledge performance, Academic skills performance, Student Satisfaction	Statistically significant difference in academic knowledge performance, academic skills performance and student satisfaction in favor of intervention group
Chao et al. (2021) [63] Taiwan	Total 45, Intervention 22, Control 23. Mean age 23.91, SD 5.5; 4th year, 14% male	Theoretical and Skills performance: Nasogastric tube feeding	Gamification	Action language, Environment, Game fiction, Immersion,	Immersive virtual environment, Scenario	3D immersive video program: Students learned through interactive video program; Digital; Individual	Traditional demonstration video	Academic knowledge performance, Student satisfaction	No statistically significant difference in academic knowledge performance, but in student satisfaction in favor of intervention group
Del Blanco et al. (2017) [64] Spain	Total 132, Intervention 62, Control 70. 2nd and 3rd year, 27% male	Skills performance: Operation theater	Serious Game	Action language, Assessment, environment, Immersion, Rules/goals	Feedback, Digital learning environment, “first person view”, Goal attainment	Video simulation game: Students played the game prior to their first experience in the operation theater; Digital; Individual	Non-access to the application	Academic skills performance	Statistically significant difference in academic skills performance in favor of intervention group
DemİRay and KeskİN Kiziltepe (2022) [50] Turkey	Total 56, Intervention 28, Control 28. 2nd year. Age 19-20 yrs., 29% male	Skills performance: CPR	Serious Game	Action language, Assessment, Conflict/challenge, environment, Game fiction, Immersion, Rules/goals	Feedback, Scoring, Adaptive challenge, Immersive virtual environment, Scenario, Goal attainment	Computer-aided game life-support: Students played a serious game prior to examination; Analogue and digital; Individual	All of the participants had theoretical lecture, video demonstration, demonstration and application on simple level CPR mannequins, but control group did not play serious game	Academic skills performance	No statistically significant difference in academic skills performance in favor of intervention group
El Machtani El Idrissi et al. (2022) [65] Morocco	Total 58, Intervention 29, Control 29. 2nd year students, 3% male	Theoretical and Skills performance: Pediatric Nursing	Serious Game	Action language, Assessment, Conflict/challenge, Environment, Game fiction, Immersion, Rules/goals	Scoring, Feedback, Adaptive Challenge, Virtual environment, Scenario, Goal attainment	Serious game: Students played online serious game with 3 scenarios with an embedded Assessment system; Digital; Individual	Traditional teaching method	Academic knowledge performance, Academic skills performance	Statistically significant difference in academic knowledge performance, however not in academic skills performance in favor of intervention group
Farsi et al. (2021) [66] Iran	Total 54, Intervention Simulation 18 (mean age 20,11, SD 1.1), Intervention serious game 18 (mean age 20, 41, SD 0.8), Control 18 (mean age 19, 78, SD 0.8). 1st semester, 61% male	Theoretical and Skills performance: CPR	Serious game	Unclear what is included in game	Unclear what is included in the game	Simulation serious game: Intervention 1: Students used a mannequin that provides feedback. Intervention 2: Used serious game on a smart-phone platform with feedback; Digital; Individual	Traditional simulation method	Academic knowledge performance, Academic skills performance	Statistically significant difference in academic knowledge performance and academic skills performance in favor of intervention group
Foss et al. (2014) [51] Norway	Total 201, Intervention 101, Control 100. Age 21; 2nd and 3rd semester, 3% male	Theoretical and Skills performance: Medical Calculation	Serious Game	Action language, Assessment, Conflict/challenge, Rules/goals	Feedback and scoring, time restraint challenge, Goal attainment	Medication game online: Played computer-based online game (a training session, self-testing section and a section of examination questions) without instructor; Digital; Individual	Traditional lectures and task solving	Academic knowledge performance	Statistically significant difference in academic knowledge performance in favor of intervention group
Fusco et al. (2021) [67] USA	Total 262, Intervention 133, Control 129. Senior year, 34% male	Theoretical and Skills performance: Acute care (sepsis), Interprofessional skills	Serious Game	Assessment, Conflict/challenge, Environment, Game fiction, Human interaction, Rules/goals	Feedback, Progression, Surprise challenges, Problems to solve, Scenario, Simulated environment, Teamwork, Goal attainment	Escape room puzzle: Escape room themed around acute management of sepsis or general acute care prior to participating in a simulated patient discharge case; Analog; Team	Escape room that included puzzles focused on general knowledge of acute care but did not relate to the theme of sepsis	Academic knowledge performance, Academic skills performance	Statistically significant difference in academic knowledge performance, however not in academic skills performance in favor of intervention group
Gu et al. (2022) [68] China	Total 154, Intervention 77, Control 77, 25% male	Skills performance: PVK	Serious Game	Action language, Assessment, Conflict/challenge, Rules/goals	Feedback, Challenge, Goal attainment	Game based mobile application: Theoretical interpretations, demonstration and opportunity to practice, and then used a game-based mobile application to practice; Digital; Individual	30 mins theoretical lecture, 30 minutes demonstration and one opportunity to practice	Academic skills performance	Statistically significant difference in academic skills performance in favor of intervention group
Gu et al. (2017) [69] China	Total 27, Intervention 13, Control 14. 2nd year, avr. age 19 yrs.	Theoretical: Fundamentals of Nursing	Gamification	Action language, Assessment, Conflict/challenge, Environment, Game fiction, Immersion, Rules/goals	Feedback and progression, Adaptive challenges, Immersive virtual environment, Scenario, Goal attainment	vSim: Virtual simulation training with 10 virtual cases in addition to traditional teaching; Digital; Individual	Traditional teaching	Academic knowledge performance, Academic skills performance	Statistically significant difference in academic knowledge performance, however not in academic skills performance in favor of intervention group
Gutierrez-Puertas et al. (2021) [70] Spain	Total 184, Intervention 92 (age mean 20.72, SD 4.83), Control 92 (mean age 20.50, SD 4.33), 52% male	Theoretical and Skills performance: CPR Life support techniques	Gamification	Action language, Assessment, Conflict/challenge, Rules/goals	Feedback, Score, challenge, competition, Goal attainment (winning)	Application life support "Guess it" (SVUAL): Playing on the application, followed by a knowledge questionnaire; Digital; Individual	Traditional 2-hours class about content followed by a knowledge questionnaire after the class and then again after 3 weeks	Academic knowledge performance	Statistically significant difference in academic knowledge performance in favor of intervention group
Ignacio and Chen (2020) [52] Singapore	Total 49, Intervention 23, Control 26. 1st year, 16% male	Theoretical and Skills performance: Pathophysiology	Gamification	Action language, Assessment, Conflict/challenge, Rules/goals	Feedback, Challenge, Competition, Time pressure, Competitive scoring – leaderboard, Goal attainment (winning)	Classroom gaming using web-based platform, kahoot.it: Case discussions followed by Kahoot; Digital; Individual	Using only case discussions	Academic knowledge performance, Academic skills performance	No statistically significant difference in academic knowledge performance, nor academic skills performance in favor of intervention group
Inangil et al. (2022) [71] Turkey	Total 70, Intervention 35 (mean age 20.95, SD 0.81), Control 35 (mean age 20.48, SD 0.78), 22% male	Theoretical: diabetes Nursing Course	Gamification	Action language, Assessment, Conflict/challenge, Rules/goals	Feedback, Challenge, Competition, Time pressure, Competitive scoring - leaderboard, Goal attainment (winning)	Powtoon and Kahoot: Animation video was shown in the beginning of the lesson followed by lecture and then question/answer over Kahoot; Digital; Individual	Traditional teaching method using PowerPoint presentation in the lecture and time for questions and answers.	Academic knowledge performance, Student satisfaction, Student motivation	Statistically significant difference in academic knowledge performance, student satisfaction and student motivation in favor of intervention group
Keys et al. (2021) [72] Canada	Total 20, Intervention 10 (mean age 22.9, SD 1.5), Control 10 (mean age 22.7, SD 1.7). Last year of studies, 10% male	Skills performance: Nursing resuscitation Education	Serious Game	Action language, Assessment, Conflict/challenge, environment, Game fiction, Immersion, Rules/goals	Progression and feedback, Immersive virtual environment, Narrative, Goal attainment	Virtual simulation game: Students practiced on basic life support and advanced cardiovascular life support first on a virtual simulation computer-based game; Digital; Individual	Students practiced on basic life support and advanced cardiovascular life support without VR simulation	Academic skills performance	Statistically significant difference in academic skills performance in favor of intervention group
Liu and Hou (2021) [73] Turkey	Total 98, Intervention 48 (mean age 20.6, SD 3.64), Control 50 (mean age 20.4, SD 3.98). Freshmen year, 23% male	Theoretical and Skills performance: Fundaments of Nursing (communication, collaboration, critical thinking)	Gamification	Assessment, Conflict/challenge, Rules/goals	Scoring, Challenge, Competition, Goal attainment (winning)	Flash cards, tabletop game and simulated clinical situations: Multi-disciplinary teaching including flash cards, tabletop games and simulated scenarios; Analogue; Individual	Traditional teaching	Academic skills performance, Student satisfaction	Statistically significant difference in academic skills performance and student satisfaction in favor of intervention group
Ma et al. (2021) [74] China	Total 104, Intervention 51 (mean age 19.22, SD 0.757), Control 53 (mean age 19.17, SD 0.802). 2nd year, 16% male	Skills performance: Disaster Nursing Competence	Serious Game	Assessment, Conflict/challenge, Game fiction, Human interaction, Rules/goals,	Feedback, Time pressure, Competition, Cooperation, Narrative, Goal attainment	Disaster themed game "Brave the wind and wave": Students received teaching through playing the game; Digital; Team	Multi-station disaster simulation	Academic skills performance	Statistically significant difference in academic skills performance in favor of intervention group
Sarvan and Efe (2022) [75] Turkey	Total 90, Intervention 45 (mean age 20.71, SD 0.84), Control 45 (mean age 20.51, SD 0.97). 5th semester, 18% male	Skills performance: Neonatal resuscitation	Serious Game	Action language, Conflict/challenge, environment, Game fiction, Immersion, Rules/goals	Challenge, Immersive virtual environment, Scenario, Goal attainment	Serious game simulation application: Theoretical training followed by the simulation with serious game; Digital; Individual	Theoretical training and video demonstration of skills	Academic knowledge performance, Academic skills performance, Student satisfaction	Statistically significant difference in academic skills performance, but not in academic knowledge performance, nor student satisfaction in favor of intervention group
Shawahna and Jaber (2020) [76] Palestine	Total 192, Intervention 94 (81 ppl with age <=20 yrs., 13 ppl >20), Control 98 (81 with age <=20 yrs., 17 >20 yrs. 2nd, 3rd or 4th year, 46% male	Theoretical: Pharmacology (of Epilepsy)	Gamification	Conflict/challenge, Rules/goals	Challenge, Goal attainment	Cross word puzzle: Received crossword puzzles as active learning tools in addition to routine learning strategy. Students could solve them as many times as they wished; Digital; Individual	Traditional teaching methods with no active learning tools	Academic knowledge performance	Statistically significant difference in academic knowledge performance in favor of intervention group
Tan et al. (2017) [77] Singapore	Total 103, Intervention 57 (mean age 21.14, SD 2.08), Control 46 (mean age 20.72, SD 0.96). 2nd year, 14% male	Theoretical and Skills performance: Blood Transfusion	Serious Game	Action language, Assessment, Conflict/challenge, environment, Game fiction, Immersion, rules goals	Feedback, Challenge, Immersive virtual environment, Narrative, Goal attainment	Serious game: Students played game on the platform 3D Hive; Digital; Individual	Traditional teaching methods and skills laboratory lessons	Academic knowledge performance, Academic skills performance	Statistically significant difference in academic knowledge performance, however not in academic skills performance in favor of intervention group
Verkuyl et al. (2017) [55] Canada	Total 47, Most students 20-25 yrs., Range 20-40. Completed 2nd year, 5% male	Skills performance: Post Operative Pediatric Nursing Course	Serious Game	Action language, Assessment, Conflict/challenge, Game fiction, Immersion, Rules/goals	Feedback, Scoring, Narrative, Learner as main character, Goal attainment	Virtual gaming simulation: Played virtual game simulation in the computer laboratory; Digital; Individual	Traditional simulation method with up to eight students under guidance of a teacher for two hours	Academic knowledge performance, Academic skills performance, Student self-efficacy	No statistically significant difference in academic knowledge performance, nor academic skills performance, nor student self-efficacy in favor of intervention group
Yildiz and Demiray (2022) [78] Turkey	Total 56 (mean age 19.62, SD 0.82), Intervention 29, Control 27, 31% male	Skills performance: IV fluid delivery	Gamification	Action language, Assessment, Control, Environment, Immersion, Rules/goals	Feedback, Scoring, Challenge, Immersive virtual reality Environment, Goal attainment	Virtual reality 3D mobile application: Students performed intravenous catheterization and fluid delivery using virtual reality followed by administration using an arm model; Both analogue and digital; Individual	Performed intravenous catheterization and fluid delivery using an IV arm model	Academic skills performance	Statistically significant difference in academic skills performance in favor of intervention group
Al-Moteri et al. (2021) [79] Saudi Arabia	Total 104, Intervention 52, Control 52. 3rd year, 0% male	Skills performance: Clinical Practice	Serious Game	Action language, Assessment Conflict/challenge, Rules/goals	Points, Levels, Challenge, Goal attainment	Rapid visual search games: Played a rapid visual search game in addition to traditional teaching method; Digital; Individual	Traditional teaching method	Academic skills performance	Statistically significant difference in academic skills performance in favor of intervention group
Astarini et al. (2018) [80] Indonesia	Total 208, 19 yrs. old. Intervention 1: 104. Intervention 2: 104, odd-semester, 11% male	Theoretical: Biochemical	Gamification	Conflict/challenge, Human Interaction, Rules/goals	Challenge, Competition, Cooperation, Goal attainment	Jigsaw and team game tournament: Intervention 1: Students played Jigsaw-like game, Intervention 2: Students learning participating in team game tournament; Analog; Both Individual and Team	None	Academic knowledge performance	Statistically significant improvement in academic knowledge performance
Bellan et al. (2017) [81] Brazil	Total 30 (10 professionals, and 20 students of 4th year)	Theoretical and Skills performance: Blood pressure	Serious Game	Assessment, Conflict/challenge, Rules/goals	Points, Competition, Goal attainment	Card game: Playing cards as a domino game with photographs of unhealthy habits and only 1 card with healthy habits; Analogue; Individual	None	Academic knowledge performance	Statistically significant improvement in academic knowledge performance
Borg Sapiano et al. (2018) [82] Malta	Total 166, 2nd and 3rd year, Mean age 22, SD 5.5, 28% male	Theoretical and Skills performance: Acute patient deterioration	Gamification	Action language, Assessment, Conflict/challenge, Environment, Game fiction, Immersion, Rules/goals	Feedback, Points, Challenge, Time pressure, Immersive virtual environment, Goal attainment	Virtual simulation program "First Act Web": The simulation with 3 scenarios (cardiac, shock, respiratory). Performance feedback was provided at the end of each scenario; Digital; Individual	None	Academic knowledge performance	Statistically significant improvement in academic knowledge performance
Butt et al. (2018) [49] USA	Total 20, Junior level. Intervention 10, Control 10, 20% male	Skills performance: Urinary Catheterization	Serious Game	Action language, Assessment, Control, Environment, Immersion,	VR-headgear and haptic gloves, Immersive virtual reality environment	Game based VR: Students used Oculus Rift head gear and haptic gloves. Wearable experience to practice catheter insertion; Digital; Individual	Practice for one hour in the simulation center supervised and with immediate feedback	Academic skills performance	No statistically significant difference in academic skills performance in favor of intervention group
Calik et al. (2022) [83] Turkey	Total 62. Majority age 22. Senior year, 8% male	Theoretical and Skills performance: Covid 19 Education Course	Serious Game	Action language, Assessment, Control, Environment, Immersion,	Level progression, Immersive virtual environment	Serious game: Playing serious game as a part of an infection and safe behavior training; Digital; Individual	None	Academic knowledge performance	Statistically significant improvement in academic knowledge performance
Chang et al. (2022) [84] Taiwan	Total 45, Intervention 21, Control 24. 1st year	Skills performance: Patient sputum suction	Serious Game	Action language, Assessment, Environment, Game fiction, Immersion, Human interaction, Rules/goals	Progression, Scenario, Immersive environment experience through video and audio, Interactive discussion, Goal attainment	Online game-based learning: Completing the watch-summarize-question learning sheets through the online game-based learning environment; Digital; Individual	Completed a paper-based learning sheet after finishing the video-based learning tasks. Participants worked on it for two weeks with 100 minutes each week	Academic skill performance, Student satisfaction, Student self-efficacy, Student motivation	Statistically significant difference in academic skill performance, student satisfaction and student self-efficacy in favor of intervention group
Chang et al. (2020) [85] Taiwan	Total 72, avr. age 21. Intervention 36, Control 36. 4th year students.	Theoretical and Skills performance: ECG Training Course	Serious Game	Action language, Assessment, Conflict/challenge, Environment, Game fiction, Immersion, Rules/goals	Gaming scores, Challenge adaptation/surprise, Immersive virtual environment, Narrative, Avatar, Goal attainment (winning)	Contextual game: Learning using an ECG-game; Digital; Individual	Traditional teaching method	Academic knowledge performance, student motivation	Statistically significant difference in academic knowledge performance and student motivation in favor of intervention group
Chau et al. (2021) [86] Hong Kong	Total 192, Senior year, 27% male	Theoretical and Skills performance: Pediatric Nursing	Gamification	Action Language, Environment, Game fiction, Immersion (no info on interactive games)	Immersive virtual environment through video-based vignette, Scenario	Technology-enhanced, inquiry-based learning program: 25 scenario-based video vignettes supplemented with critical thinking exercises, discussion guides, interactive games, reading materials, and an in-class interactive workshop; Digital; Individual	None	Academic knowledge performance	Statistically significant improvement in academic knowledge performance
Chen et al. (2015) [87] USA	Total 58 (mostly 19-21 yrs. (94.8%). Sophomore, 3% male	Skills performance: Clinical course Geriatrics	Serious Game	Action language, Conflict/challenge, Environment, Immersion, Rules/goals	Gear to simulate physical disability, Progression through stations, Surprising elements challenging them, Role playing	Aging Simulation Game: Students played an aging simulation game; Analogue; Individual	None	Academic skills performance	Statistically significant difference in academic skills performance in favor of intervention group
Cook et al. (2012) [88] United Kingdom	Total 34, Intervention 18, Control 16. 3rd year students	Theoretical and Skills performance: Life Support	Gamification	Action language, Assessment, Conflict/challenge, Game fiction Rules/goals	Feedback, Scoring, Progression, Levels, Challenge, Surprise element, Scenario, Goal attainment	PULSE (platform for under-graduate life-support education): Learning using an online platform; Digital; Individual	Traditional teaching method	Academic skills performance	Statistically significant difference in academic skills performance in favor of intervention group
Demirtas et al. (2022) [56] Turkey	Total 104 (mean age 19.18, SD 1.27), Intervention 51, Control 53. First year, 15% male	Theoretical and Skills performance: Cardiopulmonary resuscitation training	Serious Game	Action language, Assessment, Conflict/challenge, Environment, Game fiction, Immersion, Rules/goals	Feedback, Scoring, Adapting challenges, Immersive virtual environment, Scenario, Goal attainment	Serious game: Laboratory training with serious game and integrated real-time audio-visual feedback simulator; Analog and digital; Individual	Laboratory training with real time audio-visual feedback simulator	Academic knowledge performance, Academic skills performance, Student satisfaction	No statistically significant difference in academic knowledge performance, nor academic skills performance, nor student satisfaction in favor of intervention group
Englund and Basler (2021) [47] USA	Total 844 (age range 18-24 yrs.) Intervention 435, Control 409. 3rd semester, 9% male	Theoretical: Medical- surgical nursing	Gamification	Assessment, Conflict/challenge, Human interaction, Rules/goals	Feedback, Score/reward, Challenge, Risk, 1vs1 competition, Goal attainment (winning)	"Acid-base imbalance poker": Poker game with chips; Analogue; Individual	Traditional teaching method	Academic knowledge performance	Statistically significant difference in academic knowledge performance in favor of intervention group
Garcia-Viola et al. (2019) [89] Spain	Total 262, Intervention 133, Control 129, Senior year, 34% male	Theoretical and Skills performance: Basic and Advanced Life Support	Gamification	Action language, Assessment, Conflict/challenge, Human interaction, Rules/goals	Feedback, Scoring, Challenge, Competition, Goal attainment (winning)	Gamification application to learn “Basic and Advanced Life Support”: Students participated in pairs, trying to guess terms related to “Basic and Advanced Life Support” in under 30 seconds for each turn, without their partner saying the word they were trying to define; Digital; Individual	Traditional teaching method	Academic skills performance	Statistically significant difference in academic skills performance in favor of intervention group
Grech and Grech (2021) [57] Malta	Total 40, Intervention 19 (mean age 20.18, SD 1.51), Control 21 (mean age 21.35, SD 3.10), First year, 10% male	Theoretical; Public Health Education	Gamification	Action language, Assessment, Conflict/challenge, Rules/goals	Feedback, Points, Challenge, Competition, Time pressure, Competitive scoring - leaderboard, Goal attainment (winning)	Mentimeter: Student groups used webinars on Microsoft Teams followed by questions on Mentimeter; Digital; Individual	Webinar on Microsoft teams	Student satisfaction	No statistically significant difference in student satisfaction in favor of intervention group
Gutiérrez-Puertas et al. (2020) [90] Spain	Total 237, Intervention 117, Control 120. Avr. age 23, 27% male	Skills performance: Unclear	Serious Game	Assessment, Conflict/challenge, Environment, Game fiction, Human interaction, Rules/goals	Feedback, Progression, Surprising challenges, Problems to solve, Scenario, Simulated environment, Teamwork, Goal attainment	Escape room: Students had to solve tasks in escape room. They were evaluated in a 5-member team by two examiners; Analog; Team	Traditional OSCE test	Academic skills performance	Statistically significant difference in academic skills performance in favor of intervention group
Hall and Beck (2021) [91] USA	Total 76	Theoretical: Community Health Nursing Course) Intimate Partner Violence	Gamification	Assessment, Conflict/challenge, Rules/goals	Feedback, Scoring/progression, Reward to winner, Challenge Competition, Goal attainment (winning)	Storytelling and game "bingo": Presentation, Storytelling, Teaching intimate partner violence concepts in addition to PowerPoint and pictures. The 2nd intervention included also playing with bingo cards; Analogue; Individual	None	Academic knowledge performance	Statistically significant improvement in academic knowledge performance
Havola et al. (2021) [92] Finland	Total 40. 60% aged 21-25. Graduating students, 15% male	Skills performance: Clinical Reasoning skills ABCDE	Serious Game	Computer: Action language, Environment, Game fiction, Immersion VR: Action language, Conflict/challenge, Environment, Game fiction, Immersion, Rules/goals	Computer: Virtual environment, Scenario, Immersion VR: Time restrict challenge, Virtual environment, Immersion, Scenario, Immersion, Goal attainment	Computer game / simulation game and virtual reality simulation game: Intervention 1: The computer-based simulation game with nine scenarios based on clinical situations regarding surgical internal medical emergency and home care setting. Intervention 2: Virtual reality simulation with 1 resuscitation scenario. Students used VR headset HTC Vive. All students first had eight days sessions where they played computer-based simulation game at home; Digital; Individual	None	Academic skills performance	Statistically significant improvement in academic skills performance
Heinrich et al. (2012) [93] USA	Total 56 (avr. age 23.06 yrs.). Senior students, 10% male	Theoretical: Clinical Reasoning skills	Gamification	Action language, Assessment, Conflict/challenge, Environment, Game fiction, Immersion, Human interaction, Rules/goals	Feedback and progression, Adaptive challenges, Immersive virtual environment, Scenario, Goal attainment	Micro-Sim classroom-based simulation: Students used classroom simulation as a learning strategy; Analog and digital; Team	None	Academic knowledge performance	Statistically significant improvement in academic knowledge performance
Hu et al. (2021) [94] China	Total 125, Intervention 60 (mean 24.03, SD 0.74), Control 65 (mean 23.98, SD 0.70),16% male	Theoretical: Covid 19 Education Course	Serious Game	Action language, Assessment, Conflict/challenge, Game fiction, Rules/goals	Feedback and progression, Time restriction challenge, Goal attainment	Serious game-based computer learning application: Teaching about Covid19 followed by a serious game; Digital; Individual	Online lectures	Academic knowledge performance	Statistically significant difference in academic knowledge performance in favor of intervention group
Hwang and Chang (2020) [95] Taiwan	Total 56 (avr. age 20), Intervention 28, Control 28. 2nd year	Theoretical and practical; Venous Indwelling Needle Course	Serious Game	Action language, Assessment, Conflict/challenge, Environment, Game fiction, Immersion, Rules/goals	Gaming scores, Challenge; Adaptation and surprise, Virtual environment, Narrative, Avatar, Goal attainment	Game based flipped learning approach: Game-based flipped learning classroom followed by OSCE; Digital; Individual	Conventional flipped classroom learning	Academic knowledge performance, Academic skills performance, Student motivation	Statistically significant difference in academic knowledge performance, academic skills performance, and student motivation in favor of intervention group
Juwita et al. (2017) [96] Indonesia	Total 143, (avr. age 19), Freshmen, 15% male	Theoretical and Skills performance: Anatomy and physiology	Gamification	Assessment, Conflict/challenge, Human Interaction, Rules/goals	Feedback, points, Challenge, Competition, Time pressure, Competitive scoring - leaderboard, Goal attainment (winning)	Team game card tournament: Students played cards with questions on them, and the right answer would give a score point; Analogue; Team	None	Academic knowledge performance, Student motivation	Statistically significant improvement in academic knowledge performance and student motivation
Kang and Suh (2018) [97] South Korea	Total 92, Intervention 49, Control 43. 3rd year, 6% male	Theoretical and Skills performance: Chronical Illness Care	Gamification	Action language, Assessment, Conflict/challenge, Environment, Game fiction, Immersion, Rules/goals	Points (scoring), Challenge, Immersive virtual learning environment, Scenarios, Goal attainment	Smart phone-based virtual experiential application called "Care for patients with hypertensions" and "Care for patients with diabetes": Students played using the applications; Digital; Individual	Did not use applications. Rest is unclear	Academic knowledge performance, Student self-efficacy	Statistically significant difference in academic knowledge performance and student self-efficacy in favor of intervention group
Kim and Kim (2022) [98] South Korea	Total 102. Rest unspecified, 17% male	Theoretical and Skills performance: Psychiatric Nursing Course	Gamification	Assessment, Conflict/challenge, Human interaction, Rules/goals	Achievement/compensation, Challenge, Competition, Scaffolding, Role play, Goal attainment	Situation-based flipped learning and gamification: Situation-based flipped learning combined with gamification. Video lectures followed by quiz questions; Both Analogue and Digital; Individual and team	Traditional teaching method	Academic skills performance	Statistically significant difference in academic skills performance in favor of intervention group
Kinder and Kurz (2018) [99] USA	Total 98, Intervention 47 (mean age 22.48), Control 51 (mean age 21.44). Senior students, 6% male	Theoretical: Unclear	Gamification	Action language, Assessment, Conflict/challenge, Rules/goals	Feedback, points, Challenge, Competition, Time pressure, Competitive scoring - leaderboard, Goal attainment (winning)	Kahoot.it: The kahoot.it was played for in class sessions; Digital; Individual	Did not engage in Kahoot gaming strategy	Academic knowledge performance	Statistically significant difference in academic knowledge performance in favor of intervention group
Kurt and Ozturk (2021) [100] Turkey	Total 122, Intervention 64 (mean age 19.08, SD 1.17), Control 58 (mean age 19.07, SD 1.02). 1st year population, 20% male	Theoretical and Skills performance: Nursing Fundamental Course AR Injection	Gamification	Action language, Environment, Immersion	Immersive augmented reality learning environment (including visuals and animations)	Mobile augmented reality: Oral presentation and demonstration on mannequins. Students used the visuals and animations included in the application; Digital; Individual	Oral presentation and demonstration on mannequins. They were given papers explaining the process steps on the injection practices	Academic knowledge performance, Academic skills performance	Statistically significant difference in academic knowledge performance and academic skills performance in favor of intervention group
Lancaster (2014) [101] USA	Total 79, 3rd year, 12% male	Theoretical and Skills performance: Pharmacology	Serious Game	Action language, Assessment, Conflict/challenge, Environment, Game fiction, Immersion, Rules/goals	Patient simulator, Feedback and progression, Challenge, Immersive environment, Scenario, Goal attainment	High fidelity Human patient simulator and “Desire2Learn” platform: Students trained to recognize sign and symptoms of opioid overdose, while used laptops for the simulation. They used clinical judgement to individually vote on the best course of action for nursing caring for the patient; Both analogue and digital; Individual	None	Academic knowledge performance	Statistically significant improvement in academic knowledge performance
Lee Farra et al. (2015) [102] USA	Total 106, Intervention 54 (majority 18-25 yrs.), Control 52 (majority 18-25 yrs.). Senior year, 15% male	Skills performance: Placement: Decontamination Training	Gamification	Action language, Assessment, Environment, Game fiction, Immersion, Rules/goals	Microsoft Kinect, Feedback immersive virtual environment, Scenario, Goal attainment	Virtual reality simulation: Students completed simulation and then demonstrated decontamination skills using a video game control device that translates the users’ physical motions in the real world into a game or simulation environment; Analog and digital; Individual	Participants got written directions	Academic knowledge performance, Academic skills performance, Student self-efficacy	No statistically significant difference in academic knowledge performance, nor student self-efficacy in favor of intervention group. However, academic skills performance was statistically significant different in favor of control group.
Luo et al. (2021) [103] China	Total 35, 4th year, 25 female, 10 male, Avr. age 21.80, 29% male	Theoretical: Medical, Surgical, Obstetrics and Gynecology, Pediatrics, Fundamental Nursing	Gamification	Action language, Assessment, Conflict/challenge, Control, Environment, Game fiction, Immersion, Rules/goals	Feedback and progression, Adaptive challenges, Immersive virtual environment, Scenario, Goal attainment	Virtual simulation: Students used “Vsim” software with 10 simulation cases; Digital; Individual	None	Academic knowledge performance, Academic skills performance	Statistically significant improvement in academic skills performance, however not in academic knowledge performance
Maddineshat et al. (2019) [104] Iran	Total 30 (avr. age 21), 4th semester, 53% male	Theoretical: Professional Ethical Education	Serious Game	Thirteen different games utilized too many to categorize		Combination of problem-solving and gameplay for teaching bioethics: Students used games from the book “Moral games for teaching bio-ethics”. The games were used in competition style when responding to scenarios; Analogue and digital; Individual	None	Academic skills performance, Student satisfaction	Statistically significant improvement in academic skills performance, however not in student satisfaction
Marcomini et al. (2021) [46] Italy	Total 10, 2nd year. Avr age 25.70, 20% male	Theoretical: Unclear	Gamification	Action language, Assessment, Conflict/challenge, Human interaction, Rules/goals	Points, Feedback, Competition, Competitive scoring - leaderboard (graph), cooperation, Goal attainment (winning)	Unfolding case study with game elements: Unfolding case over numerous of slides on PowerPoint; Digital; Team	None	Academic skills performance	Statistically significant improvement in academic skills performance
McLafferty et al. (2010) [105] Scotland/UK	Total 100, 2nd year	Theoretical: Geriatrics	Gamification	Assessment, Conflict/challenge, Rules/goals	Feedback, Competitive Scoring, Competition, Cooperation, Goal attainment (winning)	Gaming workshops: Students competed using quiz with points that led to discussions; Analogue; Team	None	Student satisfaction	Statistically significant improvement in student satisfaction
Mitchell et al. (2021) [106] Northern Ireland, UK	Total 356, 1st, 2nd and 3rd year	Theoretical: Influenza	Serious Game	Action language, Assessment, Conflict/challenge, Rules/goals	Competition, Competitive scoring – leaderboard, Goal attainment	Influenza game "Flu Bee game": Students played online game to learn more about influenza; Digital; Individual	None	Academic knowledge performance	Statistically significant improvement in academic knowledge performance
Molina-Torres et al. (2022) [107] Spain	Total 248. 1st year, Intervention 128, Control 120, 23% male	Theoretical: Anatomy and physiology	Serious Game	Assessment, Conflict/challenge, Environment, Game fiction, Human interaction, Rules/goals	Feedback, Progression, Surprise challenge, Clues, Problems to solve, Levels, Cooperation, Scenario, Simulated environment, Goal attainment	Escape room "The Mystery of the Bodies": Students participated in teams in escape room that lasted no longer than 15 minutes; Analogue; Team	No escape room. OSCE	Academic knowledge performance	Statistically significant difference in academic knowledge performance in favor of intervention group
Mosalanejad et al. (2018) [53] Iran	Total 39	Theoretical: Psychiatric Course	Serious Game	Assessment, Conflict/challenge, Human interaction, Rules/goals	Feedback, Challenge, Cooperation, Goal attainment	Educational puzzles: The content of the course was presented through puzzle and given to small groups in combination with teamwork; Analogue and digital; Individual and Team	None	Student motivation	No statistically significant improvement in student motivation
Rachayon and Soontornwipast (2019) [108] Thailand	Total 23, 2nd year students, 1% male	Theoretical and Skills performance: Language course	Serious Game	Action language, Assessment, Conflict/challenge, Human interaction, Rules/goals	Feedback, Challenge, Cooperation, Goal attainment	Digital game in flipped learning environment: Students participated in lectures that included three step task including language learning, flipped learning, digital games; Digital; Individual	None	Academic skills performance	Statistically significant improvement in academic skills performance
Sanko et al. (2021) [109] USA	Total 395 (nursing students 318), 21% male	Skills performance: Unclear	Gamification	Assessment, Conflict/challenge, Human interaction, Rules/goals	Tracking game metrics, Challenge, Competition, Goal attainment	Friday night at the ER tabletop simulation: Simulation activity engaged teams at a board. They had to manage a busy hospital during a 24 hour period; Analog; Team	None	Academic skills performance	Statistically significant improvement in academic skills performance
Smith et al. (2018) [54] USA	Total 172, 135 (73.3% were 18-25 yrs.), 29 (15.5% were 26-34 yrs.) and 21 (11.2% were 35-50 yrs.). Senior year, 12% male	Skills performance: Decontamination Skills	Gamification	Action language, Control, Conflict/challenge, Environment, Game fiction, Immersion, Rules/goals	Challenge, Immersive virtual environment, Scenario, Goal attainment	Virtual reality simulation on immersive “Oculus Rift” developer kit 2 or on a personal computer: Immersive virtual reality simulation on head mounted display; Digital; Individual	Virtual reality on a personal computer with a mouse and a keyboard	Academic skills performance	No statistically significant difference in academic skills performance in favor of intervention group
Soyoof et al. (2022) [110] Iran	Total 160, avr. 21 yrs., 1st year students, 39% male	Theoretical and Skills performance: English for Nursing	Serious Game	Action language, Assessment, Conflict/challenge, Environment, Game fiction, Immersion, Rules/goals	Feedback, Challenge, Virtual immersive environment, Scenario, Goal attainment	Saving Lives, computer-based game: Students played the game to get familiarized with necessary skills and equipment for administrating life support while contextualizing content knowledge and specialized vocabulary; Digital; Individual	Traditional teaching method	Academic knowledge performance, Academic skills performance	Statistically significant difference in academic skills performance, however not in academic knowledge performance in favor of intervention group
Thornton Bacon et al. (2018) [111] USA	Total 164. Senior level students.	Theoretical and Skills performance: Unclear	Gamification	Assessment, Conflict/challenge, Human interaction, Rules/goals	Tracking game metrics, Challenge, Competition, Goal attainment	The Friday night at ER: Students completed the Friday night at ER while working in groups and assumed the role of a nurse leader. Their decisions affected quality, safety and costs. The game was followed by a faculty lead debriefing.; Analogue; Team	None	Academic skills performance	Statistically significant improvement in academic skills performance
Wu et al. (2020) [112] Taiwan	Total 109 (59 nursing, 50 medical interns), 15% male	Skills performance: Needle Stick	Serious Game	Action language, Assessment, Conflict/challenge, Environment, Game fiction, Immersion, Rules/goals	Feedback, Challenge; Uncertainty/surprise, Immersive virtual reality environment, Goal attainment	VR game: VR-training on needle sticking; Digital; Individual	None	Academic skills performance	Statistically significant improvement in academic skills performance
Zaragoza-Garcia et al. (2021) [113] Spain	Total 112 (mean age 22), Intervention 56, control 56; Senior year, 18% male	Theoretical and Skills performance: Medical and Surgical Courses	Gamification	Action language, Assessment, Conflict/challenge, Control, Environment, Game fiction, Immersion, Rules/goals	Feedback and progression, Adaptive challenges, Immersive virtual environment, Scenario, Goal attainment	Virtual simulation platform “VSim”: Students who had not completed 50% of their practical clinical training period during the final year received training through the “Vsim” for nursing platform. Simulations are based on high fidelity mannequins adapted for use in a virtual environment. Clinical cases with individual five clinical scenarios with program feedback and online debriefing; Digital; Individual	Students who decided to compensate their lack of practical training through a health care assisted contract	Academic knowledge performance	Statistically significant difference in academic knowledge performance in favor of intervention group
Zehler and Musallam (2021) [114] USA	Total 26. Junior level. Mean age 22, 15% male	Theoretical and Skills performance: Maternal Child Course	Gamification	Assessment, Conflict/challenge, Human interaction, Rules/goals	Feedback, Competitive scoring(points), Challenge, Competition, Cooperation in teams, Goal attainment (winning)	“Minute to Win” game, Jeopardy style: Playing minute-to-minute game, including post-partum hemorrhage stations; Analogue; Team	None	Academic skills performance	Statistically significant improvement in academic skills performance
Zwart et al. (2021) [115] Netherlands	Total 118, 8% male	Theoretical: Medication calculation	Gamification	Action language, Assessment, Conflict/challenge, Environment, Game fiction, Immersion, Rules/goals	Feedback and progression, Adaptive challenges, Avatar, Immersive virtual environment, Scenario, Goal attainment	Computer based virtual learning environment: “The Second Life” platform was used for virtual learning environment about mathematical medication in a field hospital; Digital; Individual	None	Academic knowledge performance	Statistically significant improvement in academic knowledge performance

### Gamification

The articles (*N* = 35) with gamification interventions were mostly published after 2017 (91%, *n* = 32). The most common design was quasi-experimental at 68% (*n* = 24), with 31% (*n* = 11) of these studies utilizing a control group and 37% (*n* = 13) proceeding without one. Digital interventions were the most common, used in 60% (*n* = 21) of the studies, while 29% (*n* = 10) employed analogue methods, and 11% (*n* = 4) used a combination of both.

### Digital interventions (*n* = 22)

Most of the digital intervention studies (77%, *n* = 17) reported significant positive effect on the academic achievement. Among these, 88% (*n* = 15) required only individual-, while 6% (*n* = 1) team- and another 6% (i = 1) used a combination of individual and team participation.

#### Simulation

Of the six studies (29%) incorporating simulation in their intervention [62, 63, 69, 82, 103, 113], five reported a positive effect on at least one academic achievement outcome. One reported no effect on skill performance, but significantly enhanced academic knowledge related to specific skills such as urinary catheterization [69]. Another study which included team participation in their digital simulation, reported significantly better clinical thinking ability (skill) [103]. In a study where nasogastric tube feeding skill competence was explored, they did not find any effect on student’s academic achievement, however positive student satisfaction was reported [63].

#### Augmented Reality (AR) or Virtual Reality (VR)

Four studies (18%) with only digital interventions used either AR or VR. Only one study reported using a AR-intervention [100], teaching first-year students injection techniques, both knowledge and skill performance showed significant improvements favoring intervention. Another study, examined the effect of a game-based VR-phone application [59] showed statistically significant improvement in skills performance, but no significant effect on gaining knowledge related to tracheostomy care. Two studies using VR, both evaluating the effects on gaining skills, however found no significant intervention effect [48, 54].

#### Gamified applications or platforms

Twelve studies (57%) used either a gamified application or platform in their interventions. Three of these studies examined the effect of Kahoot as a learning tool [52, 71, 99], where two of the studies reported a positive effect on knowledge performance [71, 99], student satisfaction, and student motivation [71]. Both studies were done with senior-year students. However, the last study [52] assessed Kahoot on pathophysiology first-year students, and no differences were found between the groups on knowledge or skills. Even so, all the students perceived Kahoot as a helpful tool in their learning process.

### Analogue intervention (*n* = 9)

The majority of the studies using an analog intervention 89% (n = 8) reported significant positive effect on the nursing student´s academic achievement [47, 73, 80, 91, 96, 109, 111, 114], of which 50% (*n* = 4) [96, 109, 111, 114] required team participation, followed by only individual participation 38% (*n* = 3) [47, 73, 91] and 12% (*n* = 1) [80] combined both.

#### Simulation

Three (33%) of the analogue intervention studies included simulation as a part of their intervention. One focused on communication and critical thinking, comparing traditional classroom teaching with first year students to flash cards, tabletops, and simulated clinical situations with positive effect on academic skill performance and student satisfaction [73]. The remaining two [109, 111] focused on nursing students systems thinking ability, using a tabletop simulation report significantly better academic skills performance score.

#### Other gamified educational content

The six (67%) remaining studies used different strategies or tools to gamify their educational content. A card game [47] following the mechanics of poker gaming, was created as a gamified activity on acid–base imbalance, and reported a positive effect favouring the nursing students´ academic knowledge performance in the intervention group. Similar strategies were used by the other studies, despite using other games as the foundation for their gamified educational content, such as Jeopardy [114] or bingo [91].

### Analogue and digital intervention (*n* = 4)

Seventy-five percent (*n* = 3) of the studies that used a combination of analogue and digital interventions incorporated either VR, simulation, or both. One [78], found improvement in skills, when combining physical equipment with a game-based VR-phone application, examining the effect on skills in IV fluid delivery. Another [93], used a digital simulation as a group activity, in an classroom setting and reported a significant improvement in knowledge performance. Interestingly, the last study [102], with a VR-intervention reported a negative effect on decontamination training skills with senior students in the intervention group obtaining significantly lower performance scores than the control group. There were no differences between the groups in self-efficacy nor academic knowledge performance.

#### Game attribute categories for gamification interventions

We aimed to identify if gamification interventions favored certain attribute categories or used specific combinations thereof. In our review all gamification studies (100%, *n* = 35) combined various game attribute categories, with none using only a single category. The combination of “assessment”, “challenge/conflict” and “rules/goals” are most applied together (71%, *n* = 25), either as the only attribute categories in the intervention or in combination with other game attributes (Table 6). Another prominent combination of attribute categories identified was “environment”, “game fiction” and “immersion” (29%, *n* = 10), which were identified in studies utilizing simulation as part of their intervention (Table 6).

**Table 6:**
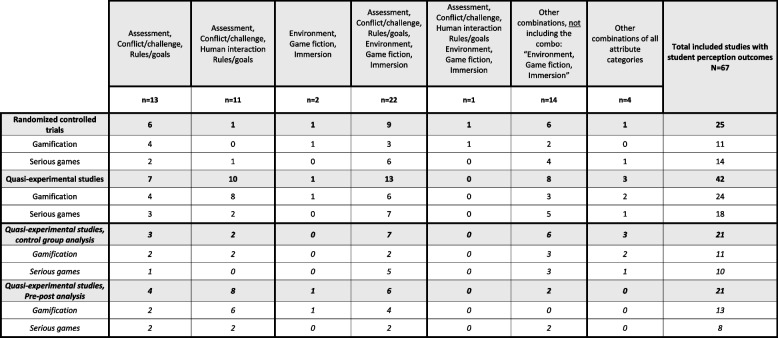
Combinations of attributes categories in gamification and serious game interventions

Interestingly, 9 of these studies included “assessment” and “rules/goals”, of which only four studies included “challenge/conflict” in the combination, contrasting with non-simulation studies where “challenge/conflict” always was paired with “assessment” and “rules/goals” (Table 6). “Action language” was a part of different combinations in 63% (*n* = 22) and the interventions are digital based. Examples of “action language” used in the interventions were, computers, mobile devices, VR-technologies, or both. For other combinations of attributes and other attributes utilized (Table 5).

### Serious game

The articles (*N* = 35), which focused on what the authors themselves referred to as SG or games”, were mostly recent publications (89% > 2017). The majority (57%) used a quasi-experimental design, with half incorporating a control group and half proceeding without one. Among the 28 studies that impacted nursing students’ academic achievement, most (71%) used digital interventions, 21% employed analogue methods, and 7% used a combination of both.

#### Digital interventions (*n* = 23)

In most of the digital intervention studies on SG, 87% (*n* = 20) reported an effect on the academic achievement. Among these, 95% (*n* = 19) required only individual participation, while team participation was required in 5% (*n* = 1).

#### Simulation

Seven (30%) of the studies with digital intervention, integrated various aspects of simulation within their respective interventions. Within this subset, the majority (71%, *n* = 5) [55, 64, 72, 74, 75] solely including simulation in the intervention, while the remaining two [49, 92] combined VR and. All seven studies aimed to enhance skill performance via the game interventions. Only two [49, 55] of the seven studies did not report any significant effect of the intervention on academic achievements.

#### Other digital SG

Among the remaining sixteen studies (70%), the majority (67%, *n* = 10) featured interventions aimed at improve knowledge performance [94, 106], either solely or in combination with skill performance [51, 65, 66, 83, 85, 95, 108, 110]. Conversely, 33% (*n* = 5) of the studies solely aimed to improve skill performance. These SG varied in aspects like design, content, and mechanics, based on their objectives or learning context, for more details (Table 5). However, despite their differences, only one study (6%) [51] out of the sixteen did not report any significant effect of the intervention on the academic achievements.

#### Analog interventions (*n* = 6)

All analog intervention studies reported significant positive effect on the academic achievement [58, 67, 81, 87, 90, 107], of which 67% (*n* = 4) required team participation [58, 67, 90, 107], followed by only individual participation 33% (*n* = 2) [81, 87].

#### Escape room

Half of the studies using analog interventions [67, 90, 107] (*n* = 3) incorporated escape room methodology. Notably, none used digital platforms, and all engaged students in teamwork. Two studies found significant difference in the academic achievement (skill [90], knowledge [107]), favoring the intervention group, after participating in the escape room. The last study [67] uniquely used an escape room in both intervention-, and control groups, differing only in theme, thus not comparing the game´s effect to a non-game element. Despite no skill performance difference, immediate recall knowledge favored the intervention group.

### Other analog SG interventions

Half of the analog intervention studies used various SG. One, examined an analog aging simulation game and found a significant skill performance improvement [87]. Another, using game cards for teaching measuring auscultatory blood pressure, reported significant increase in the knowledge performance [81]. The final study employed a Jeopardy-style serious game for ethics education, reporting significant positive increased knowledge performance and student satisfaction [58].

### Analogue and digital intervention (*n* = 6)

#### Simulation

Most studies (67%, *n* = 4) employed both analogue and digital simulation intervention to varying extent [50, 56, 60, 101]. One study uniquely combined a physical patient simulator with a serious game, reporting the only significant effect on academic achievement, increasing knowledge performance [101]. A study using a digital gaming simulation followed by classroom debriefing [60] found no significant academic achievement difference, but reported significantly higher student satisfaction and motivation in the intervention group. The remaining two studies [50, 56] found no significant results in none of the outcomes measured, when including the use of digital serious simulation games combined with physical CPR simulator [50, 56].

#### Game attribute categories for serious game interventions

In our review, combining different game attributes categories were done in 91% (*n* = 32) of the included studies on SG and none focused on only one attribute category. The combination of “assessment”, “challenge/conflict” and “rules/goals” are most applied together 66% (*n* = 21), either as the only attribute categories in the intervention or in combination with other game attributes (Table 6). Another prominent combination of attribute categories identified were “environment”, “game fiction”, and “immersion” 41% (*n* = 13), which especially were prominent in studies utilizing simulation as part of their intervention (Table 6).

### Meta-analysis

#### Meta-analysis of the academic achievement

**Fig. 3:**
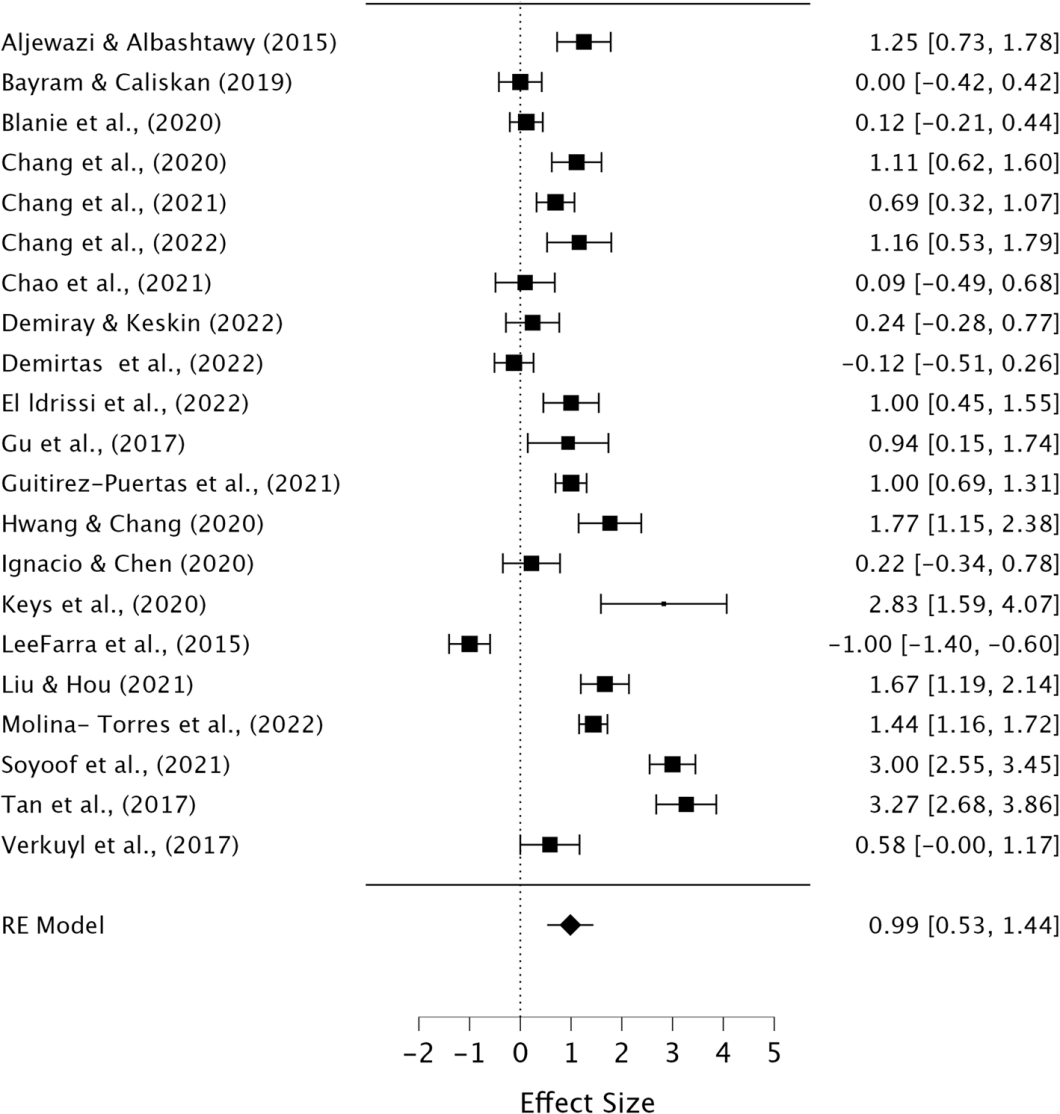
Meta-analysis of the academic achievement—forest plot

Twenty-one studies [50, 52, 55, 56, 58–60, 62, 63, 65, 69, 70, 72, 73, 77, 84, 85, 95, 102, 107, 110] provided the necessary information on the nursing students’ academic achievement to calculate the Cohens d and Standard Error (Fig. 3). The significant p-value (*p* = < *0.001)* on the Omnibus test of Model Coefficients suggest a significant impact on the nursing student’s academic achievement, with an overall effect size 0.99 [0.53, 1.44]. The significant p-value *p* = *.* < *0.001* on the Test of Residual Heterogeneity indicates heterogeneity, which is substantial as suggested by an *I*^*2*^ value of 95.01%. An insignificant Egger´s test *p* = *0.070* suggests no potential publication bias. Furthermore, the PET-PEESE test for publication bias, with a *p*-value of > 0.05, indicates no statistically significant evidence of publication bias.

#### Meta-analysis of the academic knowledge performance

**Fig. 4:**
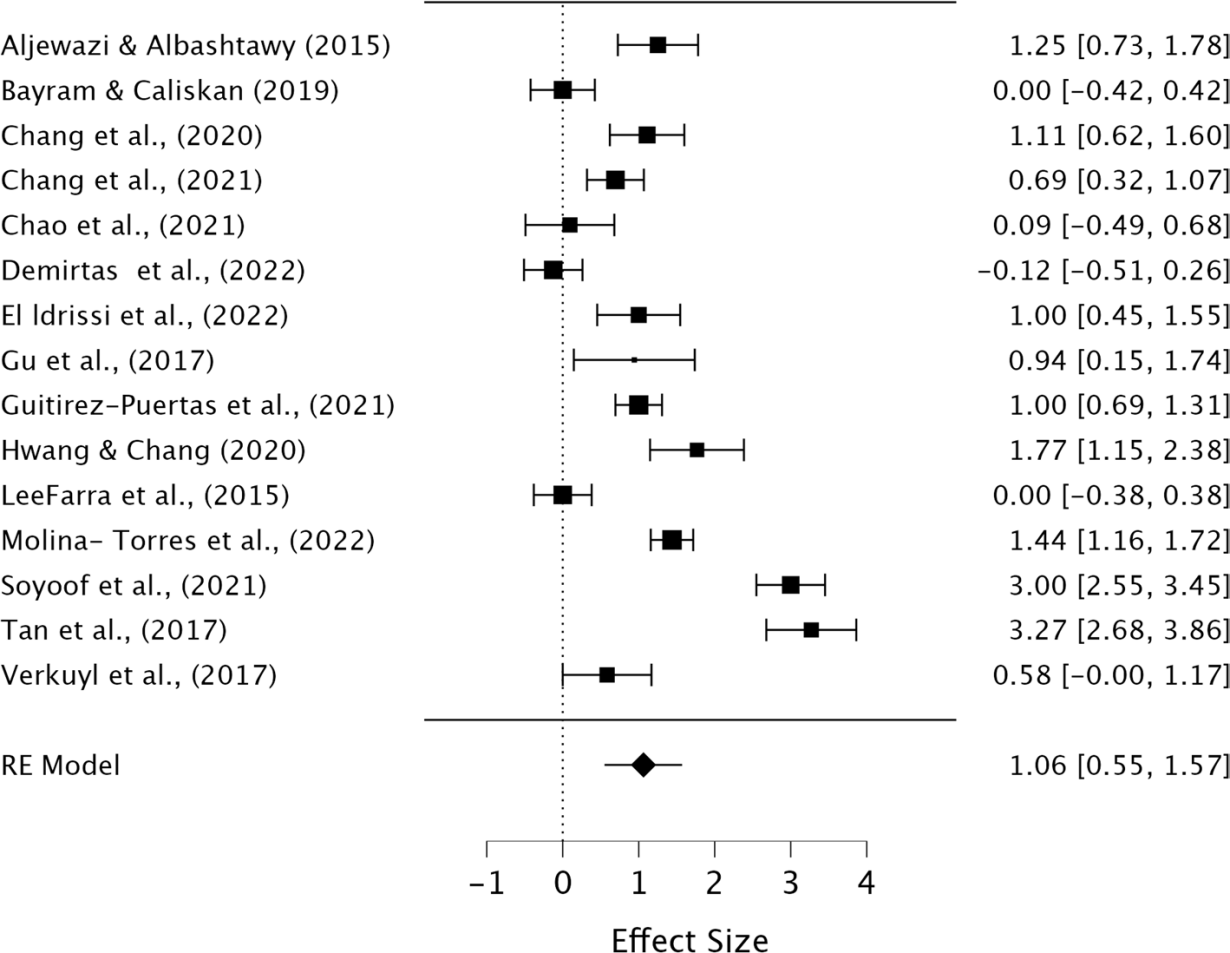
Meta-analysis of the academic achievement - forest plot

Fifteen studies [55, 56, 58, 59, 62, 63, 65, 69, 70, 77, 85, 95, 102, 107, 110] provided the necessary information on the nursing students academic knowledge performance to calculate the Cohens d and Standard Error (Fig. 4). The significant p-value *p* = < *0.001* on the Omnibus test of Model Coefficients suggest a significant impact on the nursing student’s academic knowledge performance, with an overall effect size 1.06 [0.55, 1.57]. The significant p-value *p* = *.* < *0.001* on the Test of Residual Heterogeneity indicates heterogeneity, which is substantial, as suggested by an *I*^*2*^ value of 94.95%. The Egger´s test, with an insignificant *p-value of 0.488* suggests no potential publication bias. Furthermore, the PET-PEESE to test for publication bias, with a p-value of > 0.05, indicates no statistically significant evidence of publication bias.

### Meta-analysis of the academic skill performance

**Fig. 5:**
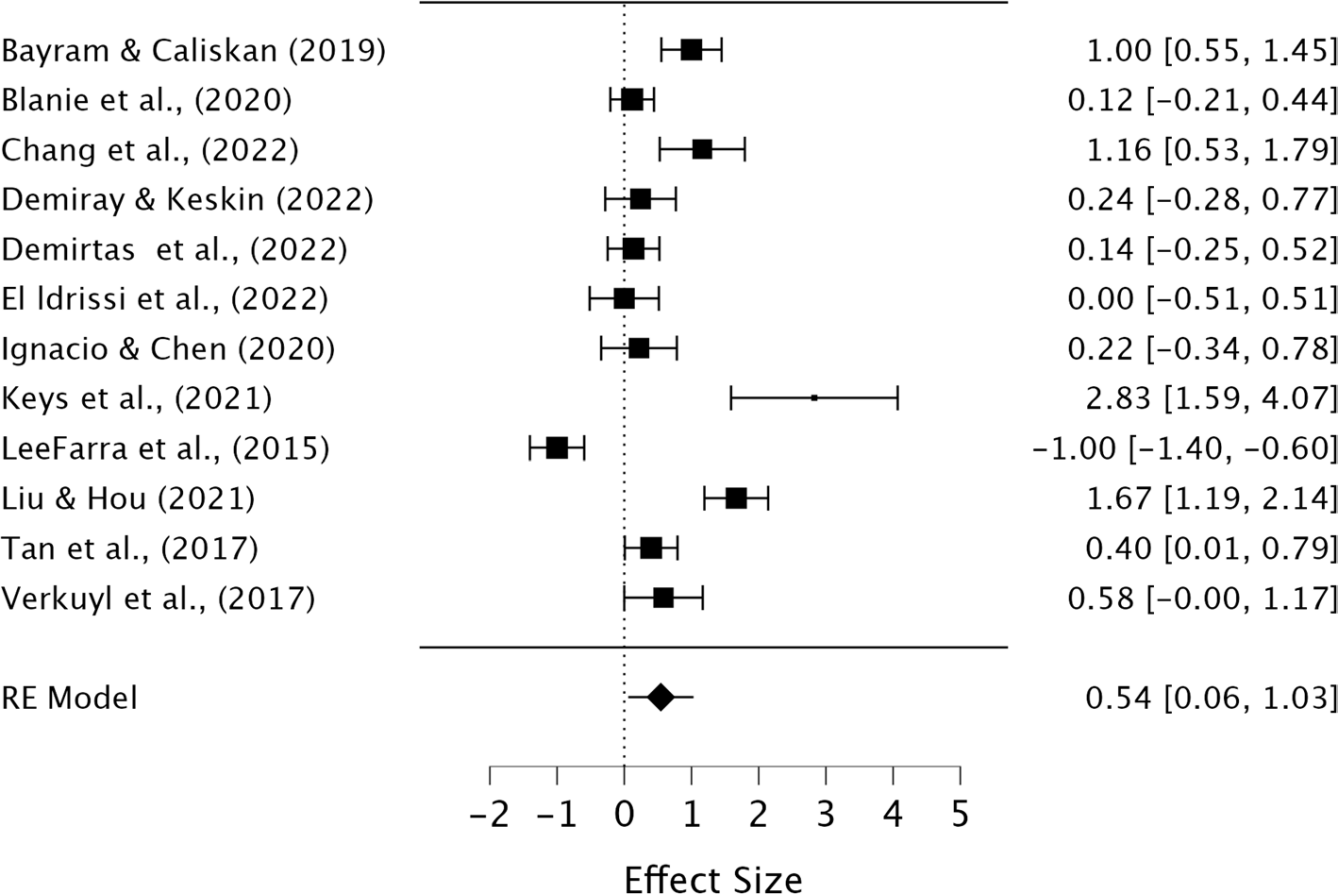
Meta-analysis of the academic skill performance—forest plot

Twelve studies [50, 52, 55, 56, 59, 60, 65, 72, 73, 77, 84, 102] provided the necessary information on the nursing students academic skill performance to calculate the Cohens d and Standard Error, and Fig. 5 show the results of the individual meta-analysis. The significant p-value *p* = *0.027* from the Omnibus test of Model Coefficients suggest a significant impact on the nursing student’s academic skill performance, with an overall effect size 0.54 [0.06, 1.03]. The significant p-value *p* = *.* < *0.001* on the Test of Residual Heterogeneity indicates heterogeneity, which is substantial as evidence by an *I*^*2*^ value of (91.78%. A significant Egger´s test (*p* = *0.003)* suggests possible publication bias. However, the PET-PEESE test for publication bias, with a *p*-value of > 0.05, indicates no statistically significant evidence of publication bias.

### Quality appraisal

The JBI quality assessment for the Randomized Controlled Trials Supplementary Material 2, showed that 10 studies were at high risk of bias due to low quality, 14 studies were at moderate risk of bias as they held a moderate quality and only two were at low risk of bias, due to their high quality.

The JBI quality assessment for the Quasi-Experimental Studies Supplementary Material 3, showed that 14 studies were at high risk of bias due to low quality, 9 studies were at moderate risk of bias as they held a moderate quality and 21 were at low risk of bias, due to their high quality.

## Discussion

### Summary of main results

Despite inconsistencies in the field, our three meta-analysis suggests that game-thinking benefits nursing education, enhancing students’ academic achievement, particularly their knowledge performance. Our narrative synthesis reveals that more than 60% of interventions in the studies were digital including either gamification or SG. Simulations are popular, frequently used in both gamified interventions and SG. The combination of “environment”, “game fiction” and “immersion” attributes, seem to be an integral part of these interventions. However, the most commonly used game attributes were “assessment”, “challenge/conflict” and “rules/goals”, probably as they constitute the gaming experience and driver in the activity [5, 16].

### Game-thinking

Most of the included studies reported effect of game-thinking on nursing students’ academic achievement. However, before delving into the impact of game-thinking on nursing education, it is important to highlight the challenges and inconsistencies presented in the evidence related to SG and gamification. Our initial focused was on gamification. However, upon examination of the available evidence, we identified certain challenges that could potentially compromise the quality of our study. To address these identified challenges, we shifted our focus to encompass game-thinking, which includes both SG and gamification.

#### Challenges in the available evidence – ambiguity and lack of consensus

The lack of a clear framework has led to subjectivity in defining SG and gamification [5, 10, 12, 13, 15, 17, 116]. Initially, we aligned with Kapp [1], viewing SG as a sub-set of gamification, formed through the gamification of traditional learning content [1]. However, it is more nuanced [5] with contrasting views [5, 10, 12, 13, 15, 17, 116]. Both concepts use the same game design elements and attributes toolkit to enhance student learning outcomes [5, 6, 13], but they differ in toolkit usage [5], intent [15] and impact on learning outcomes [5]. SG directly influence learning outcomes, often assuming the teacher role [5], while gamification affects student behavior or attitudes, such as motivation [5]. The intent behind the intervention, whether to create a game or not, may distinguish SG from gamification [15].

Research highlights challenges with inconsistent terminology in SG and gamification, including their categorization of the intervention and what constitutes game design elements [5, 7, 15, 16]. This ambiguity might lead to valuable information being overlooked in literature reviews focusing on only one of them. Despite attempts to clarify these concepts, the complexity of the evidence raises questions: Is it a serious game or gamification? Does the distinction matter?

The discussion surrounding the difference between a gamified platform or application, and a serious game has been a reoccurring theme throughout our work with this review, reflecting the lack of conceptual agreement in the evidence. The premise for these discussions was that platforms or application would be considered a game design element in the attribute category “action language”, as it provide an interface for content interaction [5, 15], often combined with other game design attributes [5]. SG, however, are full fledge games that encompass all the game design elements to various degrees, but for non-entertainment purposes [7–9]. Differentiating gamified platforms or apps from SG is challenging, with questions arising on who decides if a platform is a game or not and what criteria to use [117].

In our narrative synthesis, we included 23 studies that use simulation in their interventions, which emulate real-life scenarios for practice and learning [118]. We classified twelve studies as gamification intervention [54, 62, 63, 69, 73, 82, 102, 103, 109, 111, 113] that uses gamified digital platforms [54, 62, 63, 69, 82, 93, 102, 103, 113] or analogue activities [73, 109, 111], and eleven studies as SG [49, 50, 55, 60, 64, 72, 74, 75, 87, 92, 101]. The differentiation was challenging, as we noticed more similarities than differences when trying to differentiate these studies, which might support the use of a uniform concept such as game-thinking.

Four of our studies used web-based platforms (Kahoot [52, 71, 99] and Mentimeter [57]) to gamify their traditional teaching activities, aiming to enhance students learning outcomes [119]. These platforms, serving as an “action language”, connect players with online learning activities [5], and each educator create their own content. Students follow defined rules, track progress, compete for top scores, and aim to win, demonstrating combination of the game attribute categories “assessment”, “conflict/challenge”, “human interaction” and “rules/goals” [5, 16]. However, suppose an educator inspired by these platforms creates a quiz-game with the same functions but intends it to be a full fledge game that also includes the content rather than just an empty gaming platform, we question whether this theoretical difference impact students learning outcome.

### Nursing students and game-thinking

Our findings suggest that both serious game and gamification interventions are equally effective, indicating that their theoretical difference might not have any impact. Thus, educators might not need to choose between the two if their intervention is based on a game-thinking strategy and includes one or more of the suggested game design attributes.

The positive effects might stem from the combination of game design attributes, specifically “assessment”, “challenge/conflict” and “rules/goals”, which seem to enhance nursing students´ engagement and motivation [12]. The motivational mechanisms behind achieving goals and fulfilling needs for acknowledgment and competence appear to be central to this process [14, 28]. Both serious game and gamification also seems to enhance self-efficacy and together with engagement and motivation are linked to improvements in academic achievements [28]. Our findings suggest that incorporating game-thinking strategies as a part of educational activities can enhance nursing students´ academic engagement, self-efficacy and sustain their motivation. Further, our results suggest implementing game-thinking strategies could help retain nursing students who potentially drop-out due to academic underperformance.

Feedback on performance and recognition for their work positively impact learners’ academic achievement by fostering motivation and engagement [14]. Debriefing, central to simulation, meets these needs [118]. Studies implementing simulation, with game attributes like “environment”, “game fiction”, and “immersion” prominent, often in combination with other attributes, form a significant part of our review, and these attributes are essential in creating the virtual simulation experience [5]. A recent review reported that student learning in digital virtual simulations may depend on student facilitation and debriefing [120], highlighting the importance of assessments such as recognition and feedback.

Most interventions in our review are digital. Our results suggest that game-thinking strategies improve academic knowledge more than skill performance. Similar results are reported in another review [8]. However, game-thinking strategies have demonstrated significant positive effects on nursing students´ academic skill performance, particularly for non-physical practical training such as clinical reasoning. For physical skills like CPR-training [50, 56] or decontamination training [102] our findings show no positive [50, 56] or even negative effects [102]. One possible explanation of the difference in effect among knowledge and skill, could be explained by the more challenging learning context. Creating a gamified activity based on theoretical knowledge or cognitive skills such as clinical reasoning could be more intuitive for educators, rather than when teaching actual physical skills.

Despite overall positive impact on academic achievement, educators should apply game-thinking strategies with caution and careful planning, ensuring strategy suitability for the subject and context to avoid reducing student engagement, motivation, and academic achievement, especially if the outcome is related to academic skill performances.

### Strengths and limitations

Our study’s lies in the extensive literature search across multiple databases, though it was to English and Scandinavian languages, possibly missing relevant data in other languages. Despite Egger's test hinting at potential publication bias, statistical tests found no significant evidence of such bias. The high heterogeneity (> 90%) among the studies included in the meta-analysis and the varying quality of included studies are limitations. Another limitation is the variable quality among the included studies, as lower quality studies can exaggerate the estimate of effect. However, studies of all quality levels mostly indicate positive effects, suggesting little risk of incorrect inferences and most of the RCT´ with lower quality were rated low due to lack of blinding(S2) when following the JBI checklists. It could be discussed whether blinding is achievable, and if done is it possible to prevent unblinding. Additionally, the outcomes used in the meta-analysis is objective and as such is not as exposed to risk of bias.

Our study’ strength lies in including literature on both gamification and SG, providing a comprehensive overview of a field marked by ambiguity and lack of consensus. We acknowledge the risk of subjectivity in identifying and categorization attribute categories and elements, as they were rarely explicitly stated in the studies. We aimed to demonstrate our process in our summary table for future researchers. With half the studies originating from Asia, the results’ generalizability may be limited, though Europe and North America represents 40% of the studies.

Our approach encompasses both serious game and gamification studies, reducing the risk of overlooking relevant evidence, unlike previous research focused solely gamification [13, 15]. Given the varied definitions and similarities between the two [5, 6, 13], and the view of SG as gamification subgroup [1], we believe considering both as game thinking strategies and focusing on their attribute categories will benefit nursing education.

## Conclusion

Our research suggests that game-thinking in nursing education enhances students’ academic achievement and perceptions, especially knowledge and skill performance. However, recommending specific games or elements is challenging due to their varied use. We advocate for game-thinking strategies in future education, utilizing various game design attributes. Despite potential subjectivity in game element categorization, we believe these strategies enhance learning outcomes. We urge further research with clear frameworks and consistent terminology and call for detailed intervention descriptions.

## Supplementary Information

Supplementary Material 1.

Supplementary Material 2.

Supplementary Material 3.

## Data Availability

No datasets were generated or analysed during the current study.
