# Supplementary material for: Game-thinking; utilizing serious games and gamification in nursing education – a systematic review and meta-analysis
Source: BMC Med Educ. 2025 Jan 29;25:140. doi: 10.1186/s12909-024-06531-7 (PMC11776282; doi:10.1186/s12909-024-06531-7)
Supplement: Supplementary file 2 — Supplementary Material 2. [file 12909_2024_6531_MOESM2_ESM.docx]

**Supplementary material 2 Joanna Briggs quality assessment**

Results from Joanna Briggs appraisal checklist for Randomized Controlled Trials.

| **Author Y** | **Q1** | **Q2** | **Q3** | **Q4** | **Q5** | **Q6** | **Q7** | **Q8** | **Q9** | **Q10** | **Q11** | **Q12** | **Q13** | **JBI score** | **Quality** |
| --- | --- | --- | --- | --- | --- | --- | --- | --- | --- | --- | --- | --- | --- | --- | --- |
| (Blanié et al., 2020) | Yes | Yes | Yes | Yes | No | Yes | Yes | Yes | Yes | Yes | Yes | Yes | Yes | 12 | High  >70% |
| (Calik & Kapucu, 2022) | Yes | Yes | Yes | Yes | No | Unclear | Yes | Yes | Yes | Yes | Yes | Yes | Yes | 10 |  |
| (Bayram & Caliskan, 2019) | Yes | Yes | Yes | Unclear | Unclear | Unclear | Yes | Yes | Yes | Yes | Yes | Yes | Yes | 9 | Moderate  >50-70% |
| (Gu et al., 2022) | Yes | Yes | Yes | Unclear | Unclear | No | Yes | Yes | Yes | Yes | Yes | Yes | Yes | 9 |  |
| (Liu & Hou, 2021) | Yes | Yes | Yes | No | No | No | Yes | Yes | Yes | Yes | Yes | Yes | Yes | 9 |  |
| (Ma et al., 2021) | Yes | Yes | Yes | No | No | No | Yes | Yes | Yes | Yes | Yes | Yes | Yes | 9 |  |
| (Sarvan & Efe, 2022) | Yes | Yes | Yes | Yes | No | Unclear | Yes | Unclear | Yes | Yes | Yes | Yes | Yes | 9 |  |
| (Shawahna & Jaber, 2020) | Yes | Yes | Yes | No | No | No | Yes | Yes | Yes | Yes | Yes | Yes | Yes | 9 |  |
| (Berg & Steinsbekk, 2021) | Yes | Yes | Yes | No | No | Unclear | Unclear | Yes | Yes | Yes | Yes | Yes | Yes | 8 |  |
| (Chao et al., 2021) | Yes | Yes | Unclear | Unclear | Unclear | Unclear | Yes | Yes | Yes | Yes | Yes | Yes | Yes | 8 |  |
| (Farsi et al., 2021) | Yes | Unclear | Yes | No | No | No | Yes | Yes | Yes | Yes | Yes | Yes | Yes | 8 |  |
| (Keys et al., 2021) | Yes | Unclear | Yes | No | No | Unclear | Yes | Yes | Yes | Yes | Yes | Yes | Yes | 8 |  |
| (Verkuyl et al., 2017) | Yes | No | Yes | No | No | No | Yes | Yes | Yes | Yes | Yes | Yes | Yes | 8 |  |
| (Chang et al., 2021) | Yes | Yes | Unclear | No | No | No | No | Yes | Yes | Yes | Yes | Yes | Yes | 7 |  |
| (Foss et al., 2014) | Yes | No | Yes | No | No | No | Yes | Unclear | Yes | Yes | Yes | Yes | Yes | 7 |  |
| (Inangil et al., 2022) | Yes | Yes | Yes | No | No | No | Yes | Unclear | Yes | Yes | Unclear | Yes | Yes | 7 |  |
| (Aljezawi & Albashtawy, 2015) | Unclear | Yes | No | No | No | No | Yes | No | Yes | Yes | Yes | Yes | Unclear | 6 | Low  <50% |
| (DemİRay & KeskİN Kiziltepe, 2022) | Unclear | Unclear | Unclear | No | No | No | Yes | No | Yes | Yes | Yes | Yes | Yes | 6 |  |
| (El Machtani El Idrissi et al., 2022) | Yes | Unclear | Unclear | Unclear | Unclear | Unclear | Yes | Unclear | Yes | Yes | Yes | Yes | Unclear | 6 |  |
| (Fusco et al., 2021) | Yes | Unclear | Yes | Unclear | Unclear | Unclear | Yes | Unclear | Yes | Yes | Yes | Yes | Unclear | 6 |  |
| (Gutierrez-Puertas et al., 2021) | Unclear | Unclear | Yes | No | No | No | Yes | Unclear | Yes | Yes | Unclear | Yes | Yes | 6 |  |
| (Gu et al., 2017) | Yes | Unclear | Unclear | No | No | No | Yes | Yes | Yes | Yes | Unclear | Unclear | Yes | 5 |  |
| (Ignacio & Chen, 2020) | Yes | Yes | Yes | No | No | No | Yes | Yes | No | Yes | Unclear | No | Unclear | 5 |  |
| (Tan et al., 2017) | Yes | Yes | Yes | No | No | No | Unclear | Unclear | Unclear | Yes | Yes | Yes | Unclear | 5 |  |
| (Yildiz & Demiray, 2022) | Yes | Unclear | Unclear | No | No | No | Yes | Unclear | Yes | Yes | Yes | Unclear | Yes | 5 |  |
| (Del Blanco et al., 2017) | Unclear | Unclear | Unclear | No | No | No | Unclear | Unclear | Yes | Yes | Unclear | Unclear | Yes | 3 |  |

**Q1.** Was true randomization used for assignment of participants to treatment groups? **Q2.** Was allocation to treatment groups concealed? **Q3.** Were treatment groups similar at the baseline?
**Q4.** Were participants blind to treatment assignment? **Q5.** Were those delivering treatment blind to treatment assignment? **Q6.** Were outcomes assessors blind to treatment assignment?
**Q7.** Were treatment groups treated identically other than the intervention of interest? **Q8.** Was follow up complete and if not, were differences between groups in terms of their follow up
adequately described and analyzed? **Q9.** Were participants analyzed in the groups to which they were randomized? **Q10.** Were outcomes measured in the same way for treatment groups?
**Q11.** Were outcomes measured in a reliable way? **Q12.** Was appropriate statistical analysis used? **Q13.** Was the trial design appropriate, and any deviations from the standard RCT design
(individual randomization, parallel groups) accounted for in the conduct and analysis of the trial?
