# Supplementary material for: Game-thinking; utilizing serious games and gamification in nursing education – a systematic review and meta-analysis
Source: BMC Med Educ. 2025 Jan 29;25:140. doi: 10.1186/s12909-024-06531-7 (PMC11776282; doi:10.1186/s12909-024-06531-7)
Supplement: Supplementary file 3 — Supplementary Material 3. [file 12909_2024_6531_MOESM3_ESM.docx]

**Supplementary material 3 Joanna Briggs quality assessment**

Results from Joanna Briggs appraisal checklist for Quasi-Experimental Studies.

| **Author** | **Q1** | **Q2** | **Q3** | **Q4** | **Q5** | **Q6** | **Q7** | **Q8** | **Q9** | **JBI score** | **Quality** |
| --- | --- | --- | --- | --- | --- | --- | --- | --- | --- | --- | --- |
| (Chang et al., 2022) | Yes | Yes | Yes | Yes | Yes | Yes | Yes | Yes | Yes | 9 | High  >70% |
| (Lee Farra et al., 2015) | Yes | Yes | Yes | Yes | Yes | Yes | Yes | Yes | Yes | 9 |  |
| (Englund & Basler, 2021) | Yes | Yes | Yes | Yes | No | Yes | Yes | Yes | Yes | 8 |  |
| (Kang & Suh, 2018) | Yes | Yes | Unclear | Yes | Yes | Yes | Yes | Yes | Yes | 8 |  |
| (Soyoof et al., 2022) | Yes | Yes | Yes | Yes | Yes | Yes | Yes | Yes | Unclear | 8 |  |
| (Al-Moteri et al., 2021) | Yes | Unclear | Yes | Yes | No | Yes | Yes | Yes | Yes | 7 |  |
| (Astarini et al., 2018) | Yes | No | Yes | No | Yes | Yes | Yes | Yes | Yes | 7 |  |
| (Chang et al., 2020) | Yes | Unclear | Yes | Yes | Yes | Unclear | Yes | Yes | Yes | 7 |  |
| (Demirtas et al., 2022) | Yes | Unclear | Yes | Yes | Yes | No | Yes | Yes | Yes | 7 |  |
| (Grech & Grech, 2021) | Yes | Yes | Yes | Yes | No | Unclear | Yes | Yes | Yes | 7 |  |
| (Juwita et al., 2017) | Yes | Yes | Yes | No | Yes | Yes | Yes | Unclear | Yes | 7 |  |
| (Kim & Kim, 2022) | Yes | No | Unclear | Yes | Yes | Yes | Yes | Yes | Yes | 7 |  |
| (Kurt & Ozturk, 2021) | Yes | Unclear | Unclear | Yes | Yes | Yes | Yes | Yes | Yes | 7 |  |
| (Lancaster, 2014) | Yes | Yes | Yes | No | Yes | Yes | Yes | Yes | Unclear | 7 |  |
| (Zaragoza-Garcia et al., 2021) | Yes | Unclear | Unclear | Yes | Yes | Yes | Yes | Yes | Yes | 7 |  |
| (Zwart et al., 2021) | Yes | Yes | Yes | No | Yes | Yes | Yes | Unclear | Yes | 7 |  |
| (Chen et al., 2015) | Yes | No | No | No | Yes | Yes | Yes | Yes | Yes | 6 |  |
| (Hu et al., 2021) | Yes | Yes | Unclear | Yes | No | Yes | Yes | Yes | Unclear | 6 |  |
| (Luo et al., 2021) | Yes | Yes | Yes | No | No | Yes | Yes | Yes | Unclear | 6 |  |
| (Thornton Bacon et al., 2018) | Yes | Yes | Yes | No | Yes | Unclear | Yes | Yes | Unclear | 6 |  |
| (Zehler & Musallam, 2021) | Yes | Yes | Yes | No | Yes | Yes | Yes | Unclear | No | 6 |  |
| (Bellan et al., 2017) | Yes | NA | NA | No | Yes | NA | Yes | Yes | Yes | 5 | Moderate  >50-70% |
| (Butt et al., 2018) | Yes | No | Yes | Yes | No | Unclear | Yes | Unclear | Yes | 5 |  |
| (Chau et al., 2021) | Yes | NA | Unclear | No | Yes | Yes | NA | Yes | Yes | 5 |  |
| (Heinrich et al., 2012) | Yes | Yes | Yes | No | No | Yes | Yes | Unclear | Unclear | 5 |  |
| (Hwang & Chang, 2020) | Yes | Unclear | Yes | Yes | Yes | Unclear | Yes | Unclear | Unclear | 5 |  |
| (Kinder & Kurz, 2018) | Yes | Yes | Yes | Yes | No | Unclear | Unclear | Yes | Unclear | 5 |  |
| (Maddineshat et al., 2019) | Yes | Yes | NA | No | No | Unclear | Yes | Yes | Yes | 5 |  |
| (Sanko et al., 2021) | Yes | Yes | Yes | No | No | No | Yes | Unclear | Yes | 5 |  |
| (Wu et al., 2020) | Yes | Yes | Yes | No | No | Unclear | Yes | Yes | Unclear | 5 |  |
| (Borg Sapiano et al., 2018) | No | No | No | No | Yes | Yes | NA | Yes | Yes | 4 | Low  <50% |
| (Calik et al., 2022) | Yes | NA | NA | No | Yes | Unclear | NA | Yes | Yes | 4 |  |
| (Hall & Beck, 2021) | Yes | No | Unclear | No | Yes | Unclear | Yes | Yes | Unclear | 4 |  |
| (Marcomini et al., 2021) | Yes | Yes | Yes | No | No | Unclear | Yes | Unclear | No | 4 |  |
| (McLafferty et al., 2010) | Yes | Yes | Yes | No | No | Unclear | Yes | Unclear | Unclear | 4 |  |
| (Mitchell et al., 2021) | Yes | Yes | Unclear | No | No | Unclear | Unclear | Yes | Yes | 4 |  |
| (Rachayon & Soontornwipast, 2019) | Yes | Yes | Yes | No | No | No | Yes | Unclear | No | 4 |  |
| (Smith et al., 2018) | Yes | Unclear | Unclear | Yes | No | Yes | Yes | Unclear | Unclear | 4 |  |
| (Cook et al., 2012) | Yes | Unclear | Unclear | Yes | Unclear | Unclear | Unclear | Unclear | Yes | 3 |  |
| (Garcia-Viola et al., 2019) | Yes | Unclear | Unclear | Yes | No | No | Yes | Unclear | Unclear | 3 |  |
| (Gutiérrez-Puertas et al., 2020) | Yes | Unclear | No | Yes | No | Unclear | No | Unclear | Yes | 3 |  |
| (Havola et al., 2021) | Yes | NA | NA | No | Yes | Unclear | NA | Yes | Unclear | 3 |  |
| (Molina-Torres et al., 2022) | Yes | Unclear | Yes | Yes | No | Unclear | Unclear | Unclear | Unclear | 3 |  |
| (Mosalanejad et al., 2018) | Unclear | Unclear | Unclear | No | Yes | No | Yes | Unclear | No | 2 |  |

**Q1.** Is it clear in the study what is the ‘cause’ and what is the ‘effect’ (i.e. there is no confusion about which variable comes first)? **Q2.** Were the participants included in any comparisons similar? **Q3.** Were the participants included in any comparisons receiving similar treatment/care, other than the exposure or intervention of interest? **Q4.** Was there a control group? **Q5.** Were there multiple measurements of the outcome both pre and post the intervention/exposure? **Q6.** Was follow up complete and if not, were differences between groups in terms of their follow up adequately described and analyzed? **Q7.** Were the outcomes of participants included in any comparisons measured in the same way? **Q8.** Were outcomes measured in a reliable way? **Q9.** Was appropriate statistical analysis used?
